# Notes about morphological features of the Western Hemisphere subtribe Ardistomina, and revision of genus *Semiardistomis* Kult (Coleoptera, Carabidae, Scaritinae, Clivinini)

**DOI:** 10.3897/zookeys.210.3042

**Published:** 2012-07-24

**Authors:** Pavel Valdés

**Affiliations:** 1 Gertrudis 365 apto 5 e/ D’Strampes y Goicuría, Cp 10500, Habana, Cuba

**Keywords:** Coleoptera, Carabidae, scaritids, new species

## Abstract

Comparisons of structural features (principally mouthparts, elytral-abdominal locking mechanism, and female genitalia) of the ardistomine genera (*Aspidoglossa* Putzeys, *Ardistomis* Putzeys, and *Semiardistomis* Kult) with those features of members of the subtribe Clivinina (*Clivina* Latreille, *Oxydrepanus* Putzeys, *Schizogenius* Putzeys,*Ancus* Putzeys, *Nyctosyles* Putzeys, and *Obadius* Burmeister) confirm the taxonomic validity of the subtribe Ardistomina. Based on morphological features, the ardistomine genera are postulated to be related as follows: [*Aspidoglossa* [*Ardistomis* + *Semiardistomis*]]. Knowledge of this subtribe is further extended by taxonomic treatment of the genus *Semiardistomis* Kult. Of the 30 valid names of *Semiardistomis* previously recognized, four were proposed as variety names, and are listed as junior synonyms: *Ardistomis labialis picipes* Bates, 1881, *Ardistomis labialis nanus* Bates, 1881, and *Ardistomis labialis dilatatus* Bates, 1881; and *Ardistomis pallipes caerulea* Putzeys, 1846. Eight names, treated as specific epithets, are junior synonyms, as follows: *Ardistomis (Semiardistomis) balthasari* Kult, 1950 = *Semiardistomis glabratus* (Putzeys, 1866); *Ardistomis (Semiardistomis) emdeni* Kult, 1950 = *Semiardistomis deletus* (Putzeys, 1846); *Ardistomis aenea* Putzeys, 1866, *Ardistomis (Semiardistomis) brittoni* Kult, 1950, and *Ardistomis (Semiardistomis) marani* Kult, 1950 = *Semiardistomis flavipes* (Dejean, 1831); *Ardistomis tuspanensis* Putzeys, 1846 = *Semiardistomis labialis* (Chaudoir, 1837); *Ardistomis (Semiardistomis) vlastae* Kult, 1950 = *Semiardistomis subglabra* (van Emden, 1949); and *Ardistomis striga* Putzeys, 1866 = *Semiardistomis pallipes* (Dejean, 1831). Two new species described are *Semiardistomis exspectatus* **sp. n.** (type locality PERU, Madre de Dios, Rio Manu, 11°56'47"S, 071°17'00"W), and *Semiardistomis major* **sp. n.** (type locality PERU, Loreto, Rio Samiria, 05°12'S, 75°20'W). The 20 species of *Semiardistomis* are arranged intwo species–groups here proposed: the *puncticollis* group, including 12 species; and *labialis* group, including eight species. The species recognized are keyed, described or redescribed, and notes are provided about their Geographical distribution, habitat and activity. Distribution maps show known geographical ranges, from which are inferred patterns of speciation from a center of radiation in northern South America of both lineages.

## Abstract (es)

La comparación de las características estructurales (principalmente partes bucales, mecanismo de cierre élitro- abdominal y genitalia femenina) de los géneros ardistominos (*Aspidoglossa* Putzeys, *Ardistomis* Putzeys, y *Semiardistomis* Kult) con sus similares de miembros de la subtribu Clivinina (*Clivina* Latreille, *Oxydrepanus* Putzeys, *Schizogenius* Putzeys,* Ancus* Putzeys, *Nyctosyles* Putzeys, y *Obadius* Burmeister) confirma la validez taxonómica de la subtribu Ardistomina. Basado en características morfológicas, se postula una relación entre los géneros ardistominos de forma: [*Aspidoglossa* [*Ardistomis* + *Semiardistomis*]]. El tratado taxonómico del género *Semiardistomis* Kult extiende el conocimiento de esta subtribu. De los 30 nombres válidos previamente reconocidos, cuatro estaban propuestos como variedades, y se tratan como sinónimos: *Ardistomis labialis picipes* Bates, 1881, *Ardistomis labialis nanus* Bates, 1881, y *Ardistomis labialis dilatatus* Bates, 1881; y *Ardistomis pallipes caerulea* Putzeys, 1846. Ocho nombres usados como epítetos específicos resultan los siguientes sinónimos: *Ardistomis (Semiardistomis) balthasari* Kult, 1950 = *Semiardistomis glabratus* (Putzeys, 1866); *Ardistomis (Semiardistomis) emdeni* Kult, 1950 = *Semiardistomis deletus* (Putzeys, 1846); *Ardistomis aenea* Putzeys, 1866, *Ardistomis (Semiardistomis) brittoni* Kult, 1950, y *Ardistomis (Semiardistomis) marani* Kult, 1950 = *Semiardistomis flavipes* (Dejean, 1831); *Ardistomis tuspanensis* Putzeys, 1846 = *Semiardistomis labialis* (Chaudoir, 1837); *Ardistomis (Semiardistomis) vlastae* Kult, 1950 = *Semiardistomis subglabra* (van Emden, 1949); y *Ardistomis striga* Putzeys, 1866 = *Semiardistomis pallipes* (Dejean, 1831). Las dos especies nuevas descritas son *Semiardistomis exspectatus* **sp. n.** (localidad tipo PERU, Madre de Dios, Rio Manu, 11°56'47"S, 071°17'00"W), y *Semiardistomis major* **sp. n.** (localidad tipo PERU, Loreto, Rio Samiria, 05°12'S, 75°20'W). Las 20 especies de *Semiardistomis* se distribuyen en dos grupos de especies aquí establecidos: el grupo *puncticollis* con 12 especies y el grupo *labialis* con 8. Las especies reconocidas son incluidas en una clave de identificación, se describen o redescriben, y se dan notas a cerca de su distribución, hábitat y actividad. Los mapas de distribución muestran los rangos geográficos conocidos, de los cuales se infieren patrones de especiación a partir de un centro de radiación en el Norte de Suramérica para ambos linajes.

*N’ayant vu qu’un seul individu, j’ignore si ces différences sont constantes et si l’insecte ne constitue pas une variété de l’A. pallipes*.

(Putzeys, 1846: 646)

*Between these extremes, however, there is, either in the same or other localities, every grade of variation*

(Bates, 1881: 35)

*The species of this group are very difficult to determine without preparing a key; they form some slightly different subspecies in some localities*.

(Kult, 1950: 317)

## Introduction

Although the major part of this paper treats the species of *Semiardistomis*, a more general background is provided by a preliminary treatment of the subtribe Ardistomina and comparison with other taxa of the tribe Clivinini, principally the nominotypical subtribe Clivinina. Many scaritid genera were included in the Ardistomina, but Bousquet (2006) restricted the subtribe to the genera *Ardistomis*, *Semiardistomis* and *Aspidoglossa* Putzeys; additional evidence presented in this work supports recognition of the group.

The quotations above provide an idea of past taxonomic difficulties regarding members *Semiardistomis*. Interpretations made using non-diagnostic characters from short series of specimens have led to a taxonomic system, mostly useless, in order to identify and correlate beetles of this genus. The first arrangement of the group was made by Putzeys (1866) who recognized common characters to designate his “deuxieme groupe” of genus *Ardistomis* Putzeys. Despite that, he placed the North American species of *Semiardistomis* Kult in a separate group; this structure started recognition of the group as a different entity from its generic origin.

Kult (1950) corroborated Putzeys’ taxonomic structure, naming the “deuxieme groupe” as subgenus *Semiardistomis*, which was divided into 8 groups, and the “troisieme groupe” as subgenus *Ardistomiellus*. Kult’s structure was built using characters that proved to be superficial, so species with different origin were placed in the same group. This new scheme resulted also in inclusion of some species of the genus *Ardistomis* in *Semiardistomis*, a mistake followed by subsequent authors until Valdes (2009) made the necessary corrections.

Whitehead [in Reichardt (1977)] suggested treating *Semiardistomis* as a distinct genus, but no formal action was taken until Nichols (1988b) did so. The last published list of species names of *Semiardistomis* was compiled by Lorenz (2005).

## Material and methods

Study is based on 1038 specimens examined, provided by, or checked in, the following collections (names of curators or owners at the time of the loan in parentheses).

MHNPMuséum National d’Histoire Naturelle, Paris, France. (T. Deuve and A. Tagavian).


IRSNBInstitut Royal des Sciences Naturelles de Belgique, Bruxelles, Belgique. (A. Drumont).


CMNCanadian Museum of Nature, Aylmer, Québec, Canada. (R. S. Anderson and F. Génier).


IESInstituto de Ecología y Sistemática, Habana, Cuba. (A. Lozada).


MNHNCuMuseo Nacional de Historia Natural de Cuba, Habana, Cuba. (E. Gutiérrez).


PVCCuP.Valdés Collection, Habana, Cuba.


PBPCP. Bulirsch Collection. Prague, Czech Republic.


BMNHBritish Museum of Natural History. London, UK. (M. Barclay and C. Taylor).


HECOHope Entomological Collection, Oxford University, UK. (J. Hogan).


UASMUniversity of Alberta, Strickland Museum, Edmonton, Alberta, Canada. (G. E. Ball and D. Shpeley).


ADVAA. Dostal Collection (includes Kult Collection), Vienna, Austria.


CASDepartment of Entomology, California Academy of Sciences, Golden Gate Park, San Francisco, U.S.A. (D. H. Kavanaugh).


FSCAFlorida State Collection of Arthropods, Florida Department of Agriculture, Gainesville, Florida, U.S.A. (P. Skelley).


USNMNational Museum of Natural History, Washington, D.C., U.S.A. (T. L. Erwin).


Dissections of adults were made using standard techniques. The genitalia were preserved in glycerin in a microvial, pinned beneath the specimen from which they were removed. Mouthparts were glued on small cards pinned beneath the specimens. Observations were made using a stereobinocular microscope and a compound microscope. All line drawings were made from digital microphotographs using Corel Draw 13X.

The following measurements were made using an ocular micrometer: head length (**HL**): linear distance from apical margin of clypeus to posterior margin of right eye; length of pronotum (**PL**): linear distance from anterior to posterior margin along the midline; pronotal width (**PW**): greatest linear transverse distance; elytral length (**EL**): linear distance from basal ridge to apex along the suture; elytral width (**EW**): greatest linear transverse distance across both elytra. The standardized body length (**SBL**) is the sum of the lengths of head, pronotum and elytra. Body ratios (**PW**/**EW**, **PW**/**PL**, **PL**/**EL** and **EW**/**EL**) are given for each species. Mandible proportion is interpreted from ratio of the length of the transverse line at outer molar point (width) and length of the perpendicular distance from that line to apical edge (length).

Most terms used for structural features are found in previous works on Carabidae: Allen and Ball (1980) for adult microsculpture; Acorn and Ball (1991) for adult mandibles; Liebherr and Will (1998) for female genitalia.

## Taxonomy

### Subtribe Ardistomina

#### Diagnosis.

Within the Clivinini. the subtribe Ardistomina is characterized as follows: Elytron latero-distally (Fig. 22) with a plica (**ep**) and an adjacent abdominal (segment VII) pleural projection (**app**) that together form an elytral-abdominal locking mechanism; ovipositor (Figs 27–30) with an asetose quadranguloid laterotergite, and unsegmented gonocoxa bearing at apex few long setae.

#### Included genera.

This subtribe includes *Aspidoglossa* Putzeys,* Semiardistomis* Kult, and *Ardistomis* Putzeys.

#### Comparative morphology.

Mandibles, labium, and female genitalia (source of many diagnostic features within the tribe Clivinini) were compared within the ardistomines and between that subtribe and selected members of the nominotypic Clivinina. The latter taxon was represented by Western Hemisphere members of six exemplar genera: *Clivina* Latreille, *Oxydrepanus* Putzeys, *Schizogenius* Putzeys,* Ancus* Putzeys, *Nyctosyles* Putzeys, and *Obadius* Burmeister. Also, mandibles of *Dyschiriodes* Jeannel (subtribe Dyschiriina) were included.

**Mandibles:** Compared to those of Dyschiriina (Figs 12A–12D), the mandibles of the clivinines (Figs 9A–9D to 11A–11D) and ardistomines (Figs 6A–6D to 8A–8D) are relatively straight.

For dentition, *Dyschiriodes* exhibits the least number of occlusal teeth. The clivinine genera *Clivina* (Figs 9A–9D)and *Oxydrepanus* (Figs 10A–10D), and the ardistomine genera *Aspidoglossa* (Figs 8A–8D) and *Ardistomis* (Figs 7A–7D)have a full complement of occlusal teeth, *Semiardistomis* (Figs 6A–6D) and *Schizogenius* (Figs 11A–11D) lack the premolar tooth.

In values for L/W (a measure of relative length), the mandibles of clivinines, dyschiriines, and the ardistomine *Aspidoglossa* are relatively short (L/W 1.44–1.68), whereas the mandibles of the ardistomine genera *Semiardistomis* (L/W 1.86) and *Ardistomis* (L/W 2.25) are relatively long, with a slender terebra.

**Labium:** In form, the mentum (**m**) is markedly to slightly transverse (L/W 0.43–0.56) among the clivinine genera (Figs 17A–21A); among the ardistomine genera (Figs 13A–16A) the mentum is slightly transverse to slightly elongate (L/W 0.57–0.95), a morphocline being *Aspidoglossa—Semiardistomis—Ardistomis*.

In the Ardistomina, the apex of the lateral lobes (**ll**) is extended distally beyond the apex of the mental tooth (**mt**). This feature is varied among the clivinines: lateral lobes not extended beyond the apex of the mental tooth (*Clivina*, *Oxydrepanus*, and *Nyctosyles*, Figs 17A, 18A, 21A), to slightly extended beyond that line (*Schizogenius* and *Ancus*, Figs 19A, 20A).

The paraglossae (**pg**) are elongate, extended distinctly beyond the apex of the glossal sclerite (**gs**) in most genera of Ardistomina and Clivinina. In the ardistomine *Ardistomis* (Fig. 15B) and clivinine *Nyctosyles* (Fig. 21B) the paraglossae are much shorter than the glossal sclerite.

The glossal sclerite (**gs**) varies appreciably in both subtribes, being very large and rotund in *Semiardistomis* (Figs 13B, 14B4), broad with a broad apex in *Nyctosyles* (Fig. 21), and narrower and shorter in the remaining ardistomines and clivinines.

The glossal sclerite in the genus *Semiardistomis* has an extra pair of preapical setae (Figs 13B, 14B, **pas**). All other ardistomine and clivinine genera have a single pair of glossal setae (**as**).

Labial palpomere 3 (**lp3**) is elongate and fusiform in the Ardistomina (Figs 13A–16A). This sclerite varies in the clivinines: fusiform in *Clivina* (Fig. 17A), and variously widened in the remaining clivinine exemplar genera (Figs 18A–21A).

**Female genitalia:** The ovipositor sclerites come in two types, either ardistomine (Figs 27–30) or clivinine (Figs 31A–36A). For clivinine genera *Clivina*, *Nyctosyles* and *Obadius*, the gonocoxa is segmented, each segment designated as a gonocoxite (**gc1, gc2**). Gonocoxite 2 is more or less falcate. In ardistomine females, in contrast, the gonocoxa (**gc**) is unsegmented, and slightly curved or essentially straight. The laterotergites (**lt**) of clivinines are more or less triangular, whereas those of ardistomines are rectanguloid. Further, the laterotergites and gonocoxites of clivinines are setose along their margins, but the apex of gonocoxite 2 is glabrous. In contrast, for ardistomines, the ovipositor sclerites are essentially glabrous, but the apex of the gonocoxa bears a few setae. An unsegmented gonocoxa with reduced number of setae is also seen in Dyschiriina (Fedorenko, 1996). Within the ardistomines, the gonocoxae of *Aspidoglossa* and *Ardistomis* are moderately broad (Figs 29, 30), but those of *Semiardistomis* (Figs 27, 28) are slender, virtually rod-like.

Within the Clivinina, the reproductive tract (Figs 31A–36A) is strikingly varied, but the range of variation is about as extensive as, and similar to, that of the Ardistomina, and thus uninformative from a diagnostic perspective at subtribal level. Within each subtribe, the genera are clearly distinguishable from one another. Here, I treat only the ardistomine genera.

In *Aspidoglossa* females (Fig. 30), the spermathecal duct (**spd**) is narrow, elongate, with few loose coils proximally; the spermathecal gland (**spgd**) is relatively short. The female tract of *Ardistomis* (Fig. 29) is narrow, relatively short; the spermatheca (**sp**) is moderately long and markedly expanded distally. For *Semiardistomis* females (Figs 27, 28) the reproductive tracts of the two species-groups are sufficiently different from one another to require separate descriptions. They also differ markedly from the reproductive tracts of *Aspidoglossa* and *Ardistomis*. For details, see species-group treatments, below.

#### Habitat.

The members of the Ardistomina are hygrophilous or mesophilous, living in riparian situations, lowland swamp forests, or wet montane tropical forest.

#### Relationships.

As indicated above, of the three ardistomine genera, *Aspidoglossa* is most similar to members of subtribe Clivinina in mandibular and mental proportions and is more remote from *Ardistomis* and *Semiardistomis* in structure of the female reproductive tract than the latter two genera are from each other. In turn, *Ardistomis* and *Semiardistomis* share elongate mandibles and mentum. Based on these observations, I conclude that relationships of these three genera may be summarized using brackets as follows: [*Aspidoglossa* [*Ardistomis* + *Semiardistomis*]]

#### Geographical distribution.

This subtribe is a Western Hemisphere indigenous group. Its range extends in South America east of the Andes mountain range from central Argentina northward through Colombia, Middle America, and the West Indies to temperate North America, principally east of the Mississippi drainage basin.

### Genus Semiardistomis

Kult, 1950

http://species-id.net/wiki/Semiardistomis

Ardistomis (Semiardistomis) Kult, 1950: 301. Type species: *Clivina labialis* Chaudoir, 1837, designated by Kult (1950: 301).
Ardistomis (Ardistomiellus) Kult, 1950: 303. Type Species: *Ardistomis viridis* Say, 1823, designated by Kult (1950: 303). Synonymy established by Whitehead [in Reichardt] (1977: 392).
Semiardistomis Kult. Nichols (1988b: 98); Ball and Bousquet (2001: 45, 74); Bousquet (2006: 10); Valdés (2007: 24); Erwin (2011: 233).

#### Diagnostic combination.

**Figures 1–2.**
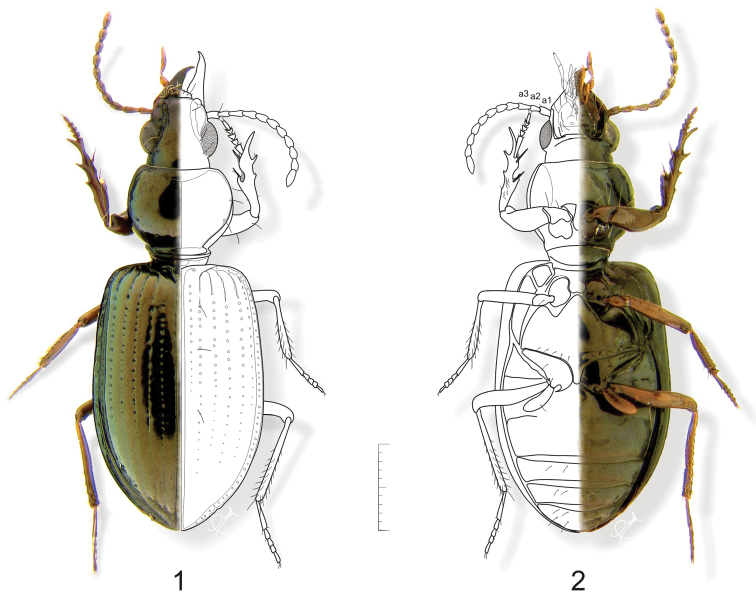
*Semiardistomis labialis* (Chaudoir). **1** Dorsal aspect **2** Ventral aspect. Legend: **a1 a2 a3 **antennomeres 1–3, respectively. Scale bar 1 mm.

Features of adults used in recognition of this genus include: antennomere 2 shorter than antennomere 3 (Fig. 1, **a2, a3**); glossal sclerite of ligula (Figs 13B, 14B) with a secondary pair of setae; mentum with median carina (Figs 13C, 14C) extended distal in form of an appendiform keel; protibia (Figs 23, 24) with basal half of ventral surface with a group of setae.

#### Description.

**Adult. Body shape, color and size** (Figs 1, 2, 37–41, 44–52): Body pedunculate. Body size small, ranging from 4.0 to 7.5 mm. Body monochromous, dark with metallic greenish and/ or brassy luster, shiny in species without microsculpture; appendages reddish brown. One species, *Semiardistomis laevistriatus* (Fleutiaux & Sallé), is dark brown a color also found in monticolous brachypterous species of the genus *Ardistomis*.

**Microsculpture:** Frontoclypeus mostly smooth; supraantennal lobes smooth; vertex with mesh pattern isodiametric; gena with mesh pattern isodiametric; gula with mesh pattern transverse. Mandibles smooth; submentum and mentum with mesh pattern isodiametric. Pronotal disc generally smooth or with mesh pattern isodiametric, microlines very shallow, marginally and smooth at the center; proepisternum smooth or with isodiametric mesh pattern, submarginal band of microsculpture absent; prosternum smooth or with mesh pattern transverse. Metasternum smooth or with mesh pattern transverse. Abdominal sterna smooth or with mesh pattern transverse. Elytra smooth or with isodiametric mesh pattern covering entirely or only part of the disc.

**Chaetotaxy:** Two pair of supraorbital setae (except for *Semiardistomis puncticollis*); pronotal disc with two pair of marginal setae (except for *Semiardistomis puncticollis* and *Semiardistomis viridis*); except for hirsute species, setae on elytral disc located in interval 3 and *Semiardistomis darlingtoni* (Kult) also in interval 5; abdominal sterna IV-VII with accessory setae.

**Head capsule:** Clypeus with anterior margin concave medially; lateral lobes distinct, projected at the same level or below anterior margin. Antennal lobes prominent, sometimes partially sulcate medially in basal half. Frontoclypeus smooth except in *Semiardistomis rugosus* (Putzeys). Frontal impressions deep and wide from base of antennal lobes to anterior margin of clypeus. Gena with sides subparallel. **Eyes:** Hemispherical, prominent. **Antennae** (Figs 1, 2): Filiform to submoniliform. Antennomere 1 (**a1**) with single preapical seta. Antennomere 2 (**a2**) with few setae, shorter than antennomere 3 (**a3**). Antennomeres 4–11 densely setose, setae short.

**Figures 3–5.**
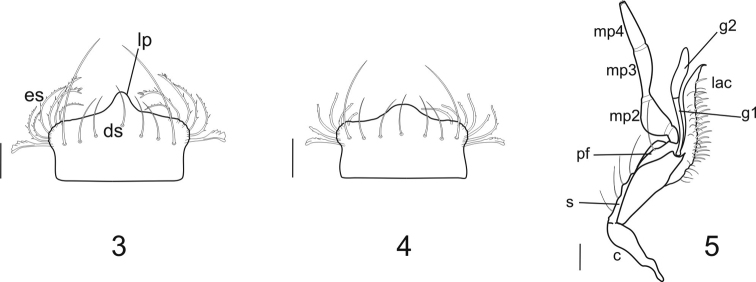
**3–4** Labrum (dorsal aspect) **3** *Semiardistomis glabratus* (Putzeys) **4** *Semiardistomis labialis* (Chaudoir) **5** RightMaxilla (dorsal aspect) of *Semiardistomis labialis* (Chaudoir). Legend : Labrum: **ds** dorsal seta **es** epipharyngeal seta **lp** labral dentiform projection. Maxilla: **c** cardo **g1** galeomere 1 **g2** galeomere 2 **lac** lacinia **mp2 mp3 mp4** maxillary palpomeres 2–4 respectively **pf** palpifer **s** stipes. Scale bar 0.1 mm.

**Figures 6–12.**
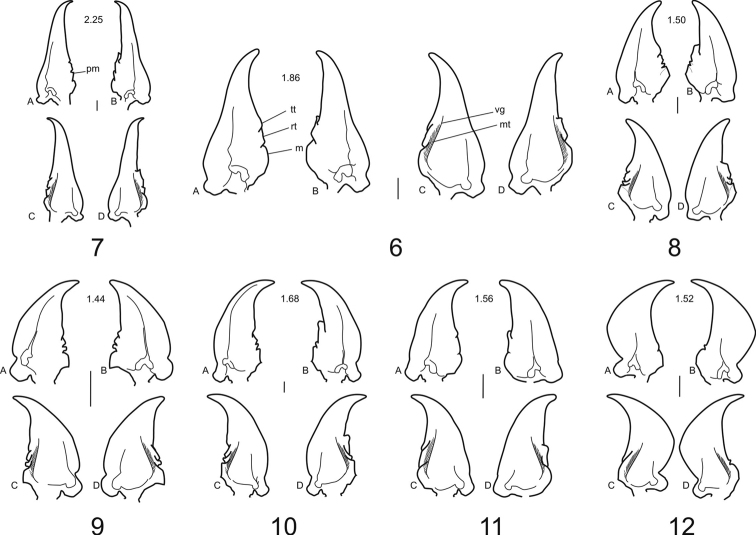
Mandibles ofArdistomina**,** Clivinina and Dyschiriina with length/width ratios: **A** left, dorsal aspect **B** right, dorsal aspect **C** left, ventral aspect and **D** right, ventral aspect) **6** *Semiardistomis labialis* (Chaudoir) **7** *Ardistomis fasciolatus* Putzeys **8** *Aspidoglossa mexicana* (Chaudoir) **9** *Oxydrepanus rufus* (Putzeys) **10** *Clivina dentipes* Dejean **11** *Schizogenius arimao* Darlington **12** *Dyschiriodes larochellei* Bousquet. Legend: Occlusal teeth: **m** molar **pm** premolar **rt** retinacular **tt** terebral. Ventral surface: **vg** ventral groove **mt** microtrichia. Scale bar 0.1 mm.

**Figures 13–21.**
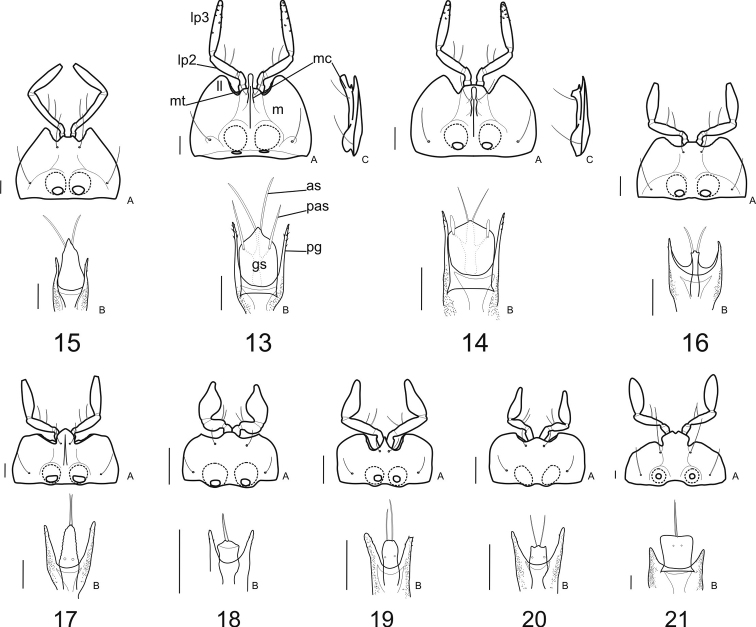
Labium ofClivinini (ventral aspects): **A** mentum and palpi **B** ligula **C** mental carina, lateral aspect. **13–14** Genus *Semiardistomis*: **13** S. glabratus (Putzeys) **14** *Semiardistomis labialis* (Chaudoir) **15*** Ardistomis fasciolatus* Putzeys **16** *Aspidoglossa mexicana* (Chaudoir) **17** *Clivina dentipes* Dejean **18** *Oxydrepanus rufus* (Putzeys) **19** *Schizogenius arimao* Darlington **20** *Ancus excavaticeps* (Putzeys) **21** *Nyctosyles planicollis* (Reiche). Legend: **as** apical setae of glossal sclerite **gs** glossal sclerite **lp2** labial palpomere 2 **lp3** labial palpomere 3 **ll** lateral lobes **mc** median carina **m** mentum **mt** mental tooth **pas** preapical seta of glossal sclerite **pg** paraglossa. Scale bar 0.1 mm.

**Mouthparts:** **Labrum** (Figs 3, 4): anterior margin angulate, dentiform medially (**dp**); dorsal setae (**ds**) 7, as for most Clivinini; apical portion of epipharyngeal setae (**es**) serrulate anteriolaterally. **Maxillae** (Fig. 5) with lacinia (**lac**) apical tooth sharp and curved; galeomere 1 (**g1**) slender, a little longer than galeomere 2 (**g2**), the latter flat, broad and sinuate in outline; palpomere 2 (**mp2**) thick and triangular in outline; palpomere 3 (**mp3**) subequal in length to palpomere 4 (**mp4**); palpomere 4 apically acuminate. Stipes (**st**) with 4 setae, palpifer (**pf**) with one seta. (This structure shows little difference from other clivinines being most similar to that of *Ardistomis*. **Mandibles** (Figs 6A–6D): elongate, about 1.9 times longer than wide; ventral groove (**vg**) moderate in length, with short microtrichia (**mt**); left mandible with terebral ridge curved, the terebral tooth (**tt**) blunt and moderate in size, visible from ventral view, retinaculum (**rt**) broad, premolar tooth (**pm**) absent, the molar tooth (**m**) broad and rounded; right mandible with terebral ridge more or less straight, the terebral tooth small, not visible from ventral view, the retinaculum with acute anterior tooth, premolar tooth absent, and the molar tooth broad and rounded. **Labium** (Figs 13A–C, 14A–C) with mental–submental suture complete; submentum with paramedian projections distinct, two pair of setae; mentum (**m**) trapezoidal, about 1.5 times wider than long; two pair of setae; lateral lobes with anterior margins angulate; mental tooth (**mt**) with distal margin almost straight, about 1/3 length of lateral lobes (**ll**); median carina (**mc**)(13C, 14C) extended distad, or not, beyond anterior margin of lateral lobes; pit organs (**po**) distinct, opened through oval orifices in basal part of mentum or on mental–submental suture; palpomere 3 (**lp3**) subequal in length to palpomere 2, palpomere 2 (**lp2**) bisetose; ligula (Figs 13B, 14B) with glossal sclerite (**gs**) notably wide, its anterior margin with acuminate median projection and two long setae (**as**), two additional small setae (**pas**) located preapically; paraglossae (**pg**) divergent from glossal sclerite and covered with microtrichia dorsally, lobes thin and acuminate, extended distally beyond distal margin of glossal sclerite, apices serrulate.

**Pronotum.** Ovate or cordate, anterior transverse and median longitudinal impressions distinct, proepisternum visible from above, lateral border not extended to base in some specimens. Anterior transverse and median longitudinal impressions distinct. Proepipleura visible from above.

**Figures 22–24.**
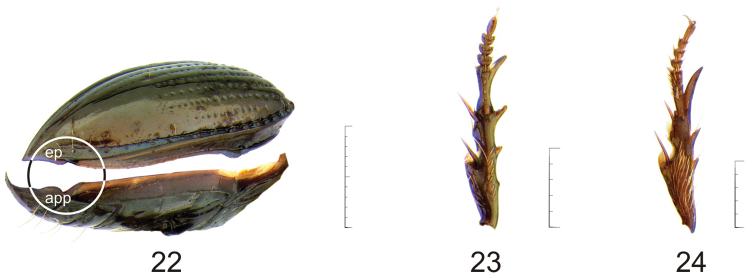
**22** Body extremity (lateral aspect) of *Semiardistomis labialis* (Chaudoir) **23–24** Protarsus (ventral aspect) of *Semiardistomis* **23** *Semiardistomis labialis* (Chaudoir) **24** *Semiardistomis glabratus* (Putzeys). Legend: **app** abdominal epipleural projectionof abdominal sternum VII **ep** elytral plica. Scale bar 1 mm.

**Elytra.** Oval, convex; striae complete or obliterated to various degrees or absent (stria 1 always complete), punctate or not, punctures shallowly to deeply impressed. Preapical epipleural plica (Fig. 22, **ep**) displaced outward, interrupting outline of elytra.

**Hind wings.** Macropterous or brachypterous.

**Underside** (Fig. 2). Metasternum with a row of setiferous punctures along margin of coxal cavity, median contact with abdomen dividing metacoxae. Suture between abdominal sterna III and IV obliterated at middle. Pleurite VII with small projection(Fig. 22, **app**) fitting into elytral epipleural plica (**ep**,).

**Legs.** Slender, protibia (Figs 23, 24) with basal half of ventral surface with a group of setae, protarsomeres in males slightly dilated.

**Figures 25–26.**
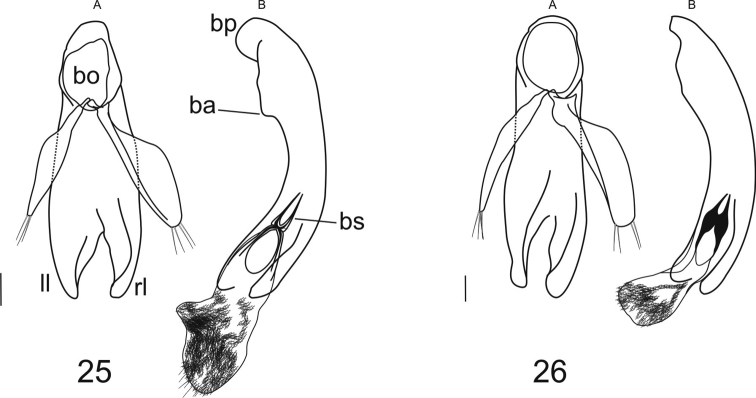
Male genitalia of genus *Semiardistomis*: **A** phallus and parameres in dorsal aspect **B** phallus and endophallus everted in lateral aspect **25** *Semiardistomis labialis* (Chaudoir) **26** *Semiardistomis glabratus* (Putzeys). Legend: **ba** basal apophysis **bo** basal opening **bp** basal projection **bs** basal sclerite **ll** left lobe **rl** right lobe. Scale bar 0.1 mm.

**Male genitalia** (Figs 25, 26). Phallus lightly sclerotized; basal bulb with basal orifice (**bo**) oval, wide; basal projection (**bp**) distinct, apical margin curved; basal apophysis (**ba**) moderate; median portion tubular; apical portion formed by two lamellae (**rl** and **ll**). Endophallus pubescent in apical portion; basal sclerite (**bs**) clearly developed. Parameres subequal in length, the right one wider, both with 4-5 apical setae.

**Figures 27–30.**
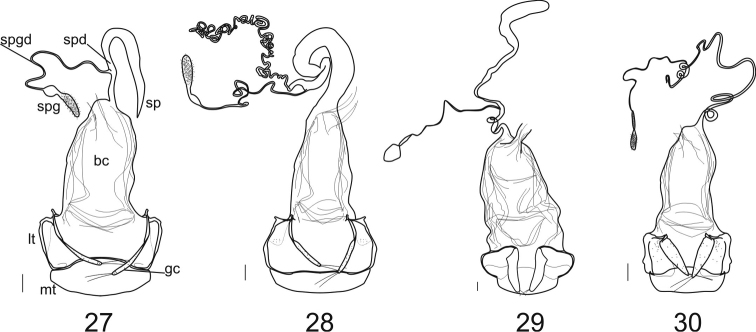
Female genitalia (ventral aspect). of Ardistomina **27** *Semiardistomis glabratus* (Putzeys) **28*** Semiardistomis labialis* (Chaudoir) **29** *Ardistomis fasciolatus* Putzeys **30** *Aspidoglossa mexicana* (Chaudoir). Legend: **bc** bursa copulatrix **gc** gonocoxa (gonocoxite 1 and 2, fused) **lt** laterotergite **mt** mediotergite **sp** spermatheca **spd** spermathecal duct **spg** spermathecal gland **spgd** spermathecal gland duct.Scale bar 0.1 mm.

**Female genitalia** (Figs 27, 28). Ovipositor: gonocoxa (**gc**) unsegmented, very slender, with a pair of setae at apex, surface with micropores; laterotergites (**lt**) thinly sclerotized, flat and asetose, sclerotized border thin and folded in basal portion with a small anterior part; mediotergite (**mt**) wide, its width delimited by external borders of laterotergites. Reproductive tract: bursa copulatrix (**bc**) elongate; spermathecal duct (**spd**) =distance from apex of bursa copulatrix to insertion point of spermathecal gland duct (**spgd**) relatively short or long, spermathecal gland duct elongate; spermatheca (**sp**) various in thickness, length, and conformation; see description of this structure for each of the two species-groups.

#### Morphological notes.

**Cuticular sculpture:** The absence of a submarginal band of microsculpture on the proepisternum has been used to differentiate *Semiardistomis* from *Ardistomis* but the character state is not diagnostic since the band has been lost in at least two lineages of *Ardistomis* (Valdes, 2009).

The microsculpture on the elytral disc has a diagnostic value, the absence of microsculpture being the apotypic state.

**Vestiture:** Hirsuteness is a common phenomenon in this genus. In the two species groups, this apotypic character state appears, its origin independently developed. In the *puncticollis* group, three species have hirsute structures: *Semiardistomis puncticollis* from southern U.S.A. (supraorbital area of the head capsule, pronotal disc, elytral disc, profemur and abdominal sternum VII), *Semiardistomis pilosellus* (Kult) from southern South America (elytral disc and profemur) and *Semiardistomis subglabra* (van Emden) from South America (profemur). In the *labialis* group, two species have hirsute structures: *Semiardistomis viridis* from southern U.S.A. (pronotal disc and elytral disc) and *Semiardistomis propinquus* (Putzeys) from Mexico (elytral disc),

**Elytra:** In this genus, I regard the state of having the striae complete and impunctate as plesiotypic, being present in both species groups.

**Hind wings:** Macropterous except *Semiardistomis laevistriatus* brachypterous. Nichols (1988a) also reported that *Semiardistomis puncticollis* is brachypterous.

**Legs:** Protarsomeres in males so slightly dilated that this feature is useless for sex recognition.

**Male genitalia:** Phallic structure enhances definition of the two species groups of *Semiardistomis*. See details, provided in the species-group diagnoses. The differences in the phallic structures are too small to separate the species within each group, a situation also known in other clivinine genera, such as *Schizogenius* Putzeys (see Whitehead 1972).

#### Larvae.

The first instar of *Semiardistomis* was described by Bousquet (2006). It was compared with *Ardistomis* larva by Valdes (2007) who pointed out two notable synapotypic features: absence of the coronal suture and a segmented maxillary palpomere 4.

#### Classification.

Based primarily on male and female genitalic features, the species of *Semiardistomis* are arranged in two species-groups designated as the *puncticollis* and *labialis* group, respectively.

#### Criteria for species definition.

Of the 20 known species of *Semiardistomis* only those occurring in U.S.A. (*Semiardistomis viridis* (Say) and *Semiardistomis puncticollis* (Dejean)) are represented by large series of specimens in collections (Nichols 1988a; Bousquet 2006). The species inhabiting Central and South America and the West Indies are represented in collections by few specimens which give only an incomplete picture of the ranges of the species.

Members of this genus are difficult to identify in part because of a shortage of useful external features, and in part because the male and female genitalia, so useful in distinguishing between species groups, are not useful within each group. Moreover, some of the characters traditionally used to separate the species have been found in large samples to vary extensively. Some samples are a mixture of combinations from extreme patterns considered as different species; being impossible to demonstrate if those populations are hybrids or simply polymorphs, while in other putative species, allopatric populations exhibit no evident differences. In the *labialis* group in southern South America, the species limits are especially difficult to assess probably because the group is young and composed of species with a great power of dispersal. Without direct evidence of the state of gene flow between known populations, it is almost impossible to testify in favor of the reproductive isolation of each species described or in other cases to designate subspecies. I will follow a criterion with a practical value, so maybe for some species, taxonomic determination will denote unintentionally a combination of extreme characters shared by more than one biological species.

#### Geographical distribution.

(Figs 57–59)The range of this genus is co-extensive with the range of the Ardistomina.

**Figures 31–36.**
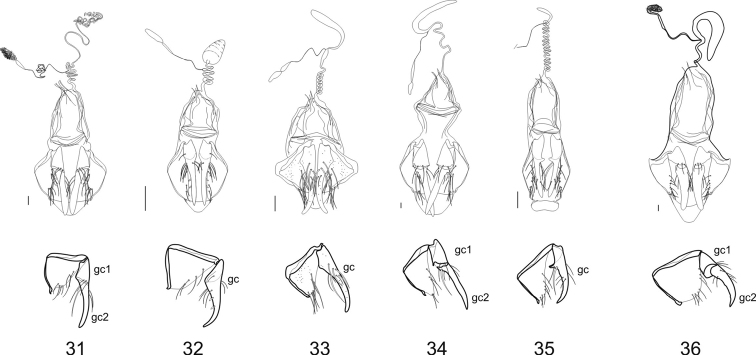
Female genitalia (dorsal aspect) and gonocoxites /laterotergites (lateral aspect) of Clivinina **31** *Clivina dentipes* Dejean **32** *Oxydrepanus rufus* (Putzeys) **33** *Schizogenius arimao* Darlington **34** *Nyctosyles planicollis* (Reiche) **35** *Ancus excavaticeps* (Putzeys) **36** *Obadius insignis* Burmeister. Legend: See legend for **Figs** **27–30**, except gonocoxite 1 (**gc1**) and. gonocoxite 2 (**gc2**) Scale bar 0.1 mm.

#### Key to described species of *Semiardistomis*, based on adult characters

**Table:** 

1	Elytral disc plurisetose	2
1’	Elytral disc with setiferous punctures in interval 3, or sometimes also in interval 5	5
2(1)	Profemur plurisetose	3
2’	Profemur with the usual setae	4
3(2)	Pronotal disc plurisetose (along margin and on disc). Habitus as in Fig. 48. USA	*Semiardistomis puncticollis*(Dejean*)*
3’	Pronotal disc with the usual 2 pairs of marginal setae. Habitus as in Fig. 44. South America	*Semiardistomis pilosellus* (Kult)
4(2’)	Pronotal disc plurisetose along margin. Habitus as in Fig. 55. USA	*Semiardistomis viridis* (Say)
4’	Pronotal disc with the usual 2 pairs of marginal setae. Habitus as in Fig. 54. Mexico	*Semiardistomis propinquus* (Putzeys)
5(1’)	Elytron with two setiferous punctures on interval 3; humerus rounded; brachypterous. Habitus as in Fig. 47 Guadeloupe	*Semiardistomis laevistriatus* (Fleutiaux & Sallé*)*
5’	Elytron with three or more setiferous punctures on interval 3; macropterous	6
6(5’)	Elytron with three setiferous punctures on interval 3	7
6’	Elytron with more than three setiferous punctures on interval 3	12
7(6)	Elytral striae impunctate. Habitus as in Fig. 52. South America	*Semiardistomis pallipes* (Dejean)
7’	Elytral striae punctate	8
8(7’)	Elytral disc with isodiametric mesh pattern	9
8’	Elytral disc smooth	10
9(8)	Elytral striae impressed only in basal fifth, punctures deep along three-quarters of elytra, surface completely covered with isodiametric mesh pattern. South America	*Semiardistomis deletus* (Putzeys)
9’	Elytral striae obliterated in apical third, punctures moderately deep, surface with isodiametric mesh pattern, evanescent in central area of elytral disc in some specimens. South America	*Semiardistomis flavipes* (Dejean)
10(8’)	Elytral striae obliterated in apical fifth, punctures moderately deep. Habitus as in Fig. 51. West Indies	*Semiardistomis cyaneolimbatus* (Chevrolat)
10’	Elytral striae impressed only in basal fifth	11
11(10’)	Elytral punctures deeply impressed in basal two-thirds of elytra. Habitus as in Figs 1, 2. Central America	*Semiardistomis labialis* (Chaudoir)
11’	Elytral punctures moderately impressed in basal third of elytra. Southern South America	*Semiardistomis semipunctatus* (Dejean)
12(6’)	Elytral disc surface completely covered with isodiametric mesh pattern. Habitus as in Fig. 46. South America	*Semiardistomis maindroni* (Kult)
12’	Elytral disc surface smooth	13
13(12’)	Elytral striae complete; impunctate	14
13’	Elytral striae incomplete	17
14(13)	Front between antennal lobes with transverse rugae. Habitus as in Fig. 50. Southern Brazil	*Semiardistomis rugosus*
14’	Front between antennal lobes smooth	15
15(14’)	Elytron with five setiferous punctures on interval 3; body size more than 7 mm. Habitus as in Fig. 49. Peru	*Semiardistomis major* n. sp.
15’	Elytron with four setiferous punctures on interval 3; body size less than 6 mm	16
16(15’)	Pronotum markedly cordate, 1.25 times wider than long; humeri square, sides subparallel. Habitus as in Fig. 37. Colombia	*Semiardistomis cordicollis* (Putzeys)
16’	Pronotum subcordate, 1.14 times wider than long; shoulders rounded. Habitus as in Fig. 38. South America.	*Semiardistomis exspectatus* n. sp.
17(13’)	Elytron with eight setiferous punctures in interval 3	18
17’	Elytron with four setiferous punctures in interval 3	19
18(17)	Elytron with three setiferous punctures in interval 5. Habitus as in Fig. 39. Brazil	*Semiardistomis darlingtoni* (Kult)
18’	Elytron with interval 5 asetose. Habitus as in Fig. 40. South America	*Semiardistomis subglabra* (van Emden)
19(17’)	Elytral striae 3,4,5 and 6 impressed in basal half of disc, stria 2 present. Body length around 4 mm. Habitus as in Fig. 45. South America	*Semiardistomis jedlickai* (Kult)
19’	Elytral striae 3, 4, 5 and 6 impressed in basal third of disc, stria 2 absent. Body length around 6 mm. Habitus as in Fig. 41. South America	*Semiardistomis glabratus* (Putzeys)

## The puncticollis species–group

This group is defined by the following: mentum with median carina (Fig. 13C) extended slightly distad anterior margin of lateral lobes (except *Semiardistomis puncticollis* with this structure (putatively) reduced); mental pit organs opened through oval orifices on the mental–submental suture. Males with wide phallus, undefined basal bulb (Fig. 26B) and a prominent, thickly sclerotized endophallic basal sclerite (Fig. 26A).

Female reproductive tract (Fig. 27; cf. Fig. 28) with spermatheca duct (**spd**) relatively short; spermatheca (**sp**) bent in its middle portion, wider from this point and with acute apex.

### Included species

*Semiardistomis cordicollis* (Putzeys, 1846)

*Semiardistomis darlingtoni* (Kult, 1950)

*Semiardistomis exspectatus* sp. n.

*Semiardistomis glabratus* (Putzeys, 1866)

*Semiardistomis jedlickai* (Kult, 1950)

*Semiardistomis laevistriatus* (Fleutiaux & Sallé, 1889)

*Semiardistomis maindroni* (Kult, 1950)

*Semiardistomis major* sp. n.

*Semiardistomis pilosellus* (Kult, 1950)

*Semiardistomis puncticollis* (Dejean, 1831)

*Semiardistomis rugosus* (Putzeys, 1866)

*Semiardistomis subglabra* (van Emden, 1949)

#### Semiardistomis cordicollis

(Putzeys, 1846)

http://species-id.net/wiki/Semiardistomis_cordicollis

Figs 37 57

Ardistomis cordicollis Putzeys, 1846: 646; Lorenz 2005: 146.
Ardistomus cordicollis Putzeys: Csiki 1927: 547; Blackwelder 1944: 27.
Semiardistomis cordicollis (Putzeys); Erwin 2011: 233.

##### Type material.

Holotype at MHNP, pinned, labeled: handwritten “Lectotype Ardistomis cordicollis Putz. By Erwin 1976”/ “Lectotype Clivina labialis Chd. Des. S.W. Nichols 1984”/ printed in red paper “lectotype”/ handwritten in box “cordicollis Putzeys. Nlle Grenade C. Reiche”.

##### Type area.

Given by Putzeys (1866: 646) as “Nouvelle Grenade” = Colombia.

##### Diagnosis.

Elytral surface smooth, striae complete, impunctate, 4 setiferous punctures in interval 3, humeri square, sides subparallel. Pronotum notably cordiform. Size given by Putzeys as 4.5 mm.

##### Habitus.

Fig. 37.

##### Note.

The unique type seen appears to be a member of the *puncticollis* group. I have been unable to examine the genitalia, but the general structure is similar to the species of this group (I observed 4 setiferous punctures in interval 3 contrary to Putzeys’ original description which mentions 3 punctures). The species appears to be most similar to *Semiardistomis exspectatus* sp. n.

##### Geographical distribution

(Fig. 57). This species is known only from an unspecified location in Colombia.

**Figures 37–38.**
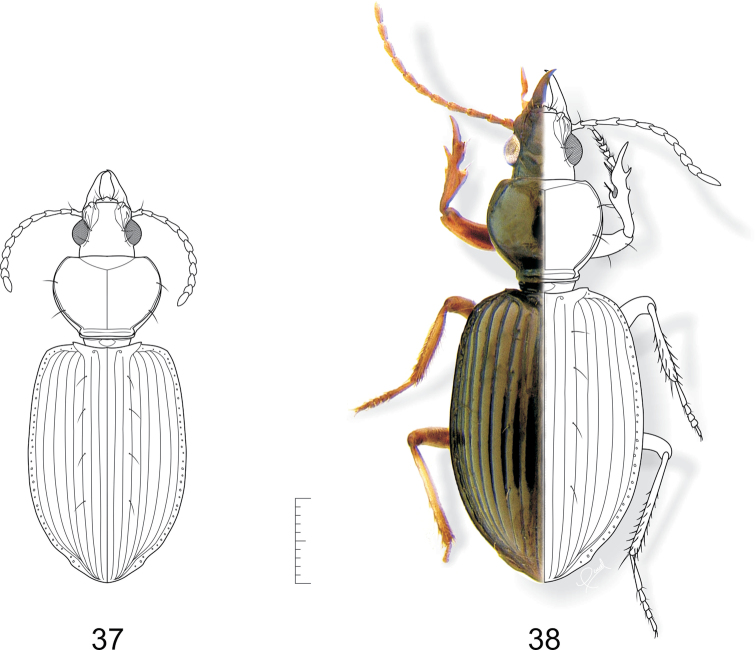
**37** *Semiardistomis cordicollis* (Putzeys) **38** *Semiardistomis exspectatus* sp. n. Dorsal aspect. Scale bar 1 mm.

#### Semiardistomis darlingtoni

(Kult, 1950)

http://species-id.net/wiki/Semiardistomis_darlingtoni

Figs 39 57

Ardistomis (Semiardistomis) darlingtoni Kult, 1950: 311.
Semiardistomis darlingtoni (Kult); Lorenz 1998: 136.

##### Type material.

Holotype male (ADVA), glued on pinned card, genitalia attached in microvial, labeled: handwritten “darlingtoni 57 Det. K. Kult”/ “Darlingtoni Kt. det. K.Kult”/ “ Brasilia Jatahy (Goyas)”/ printed on red paper “TYPE”.

##### Type area.

Given by Kult (1950: 312) as “Brasilia, Goyaz”, Brazil

##### Diagnosis.

Elytral surface smooth, impunctate, elytral striae impressed in basal third, three setiferous punctures on interval 5 and 8 in interval 3, stria 2 absent, prominent humeral tooth at junction of third stria with marginal channel. Profemur glabrous. Abdominal sternum VII with 5+5 setiferous punctures.

##### Habitus.

**Figures 39–40.**
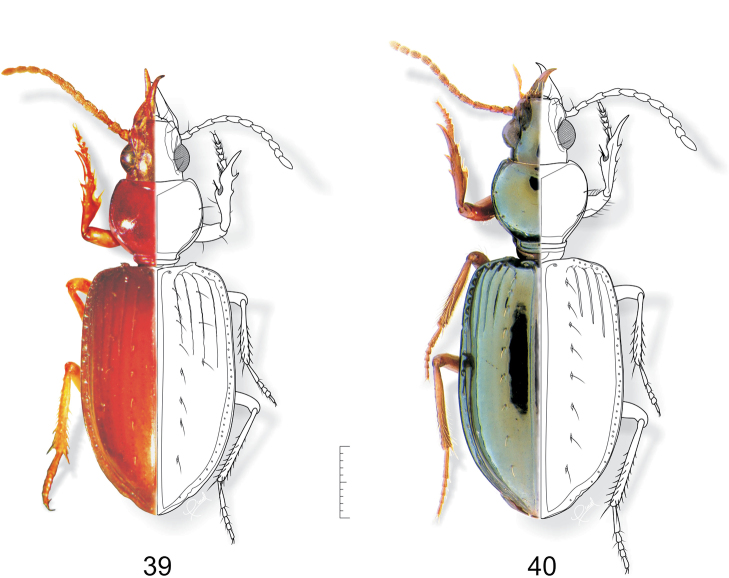
**39** *Semiardistomis darlingtoni* (Kult) (teneral) **40** *Semiardistomis subglabra* (van Emden). Dorsal aspect. Scale bar 1 mm.

dorsal aspect, as in Fig. 39.

##### Measurements and variation.

Variation of measurements (mm) and ratios for *Semiardistomis darlingtoni* (n=1) are: HL = 0,70; PL = 1,22; PW = 1,38; EL = 3,42; EW = 2,21; SBL = 5,4; PW/EW = 0,62; PW/PL = 1,13; PL/EL = 0,36; EW/EL = 0,64.

##### Geographical distribution

(Fig. 57)**.** Known only from the type area.

##### Note.

The holotype is teneral. The species is closely related to *Semiardistomis subglabra*.

#### Semiardistomis exspectatus sp. n.

urn:lsid:zoobank.org:act:1C3A9901-2441-4E08-9860-9DBE99ADBA6F

http://species-id.net/wiki/Semiardistomis_exspectatus

Figs 38 57

##### Holotype.

Male (USNM), glued on pinned point, genitalia attached in microvial, labeled: printed "Peru: Madre de Dios Rio Manu, BIOLAT Biol. Sta. Pakitza, 356 m, 24 June 1993 11°56'47"S, 071°17'00"W T. L. Erwin & F. Pfuno"/ "Treading in leaf litter half buried in quick- silt at edge of Que. Trepanatrunco Tr. Tachigali"/ "135 Lot 510"/ "BIOLAT/ COLE 000019005".

##### Paratypes.

19 exemplars (USNM), 2 exemplars (PVCCu) labeled same as the holotype. 14 exemplars (USNM) labeled: printed “Peru: Madre de Dios Rio Manu, BIOLAT Biol. Sta. Pakitza, 356 m, 11 June 1993 11°56'47"S, 071°17'00"W T. L. Erwin & F. Pfuno"/ "Tr. Castañal/ 12.5 Que. Paujil on sand among small stones near stream edge at night Lot 437".

##### Type locality.

PERU, Madre de Dios, Rio Manu, 11°56'47"S, 071°17'00"W.

##### Etymology.

After have a picture of zoogeographic patterns for some characters from the rest of described species of *Semiardistomis*, appearance of this new form filled a gap in the scenario of the group *puncticollis* . So the specific epithet derived from Latin adjective, meaning expected.

##### Diagnosis.

Elytral surface smooth, striae complete, impunctate, 4 setiferous punctures on interval 3, shoulders rounded. Profemur glabrous. Abdominal sternum VII with 4+4 setiferous punctures.

##### Habitus.

Fig. 38.

##### Measurements and variation.

Variation of measurements (mm) and ratios for *Semiardistomis exspectatus* **sp.n.** (n=10) are: HL = 0,64–0,68–0,74; PL = 1,09–1,18–1,28; PW = 1,28–1,35–1,44; EL = 3,20–3,37–3,68; EW = 2,14–2,32–2,66; **SBL =** 4,93–5,24–5,70; PW/EW = 0,58; PW/PL = 1,14; PL/EL = 0,35; EW/EL = 0,69.

##### Description.

Body piceous with green reflections, mouthparts and antennae testaceous, legs ferrugineous.

Frons, gena, antennal lobes, pronotum, proepisternum and elytra smooth. Prosternum with microsculptural mesh pattern isodiametric. Metasternum and abdominal sterna with microsculpture in form of a shallow transverse mesh pattern.

Anterior marginal setae on pronotal disc equidistant between anterior angles and posterior setae. Elytral disc with 4 setae in interval 3. Abdominal sternum VII with ambulatory setae near base, 2 on each side; inner pair of preapical setae equidistant each other. Profemur glabrous. Ventral surface of protibia with many setae on basal half.

Clypeus with anterior margin concave medially; lateral lobes distinct, projected at the same level of anterior margin. Frontal impressions deep and wide. Supraantennal lobes with median sulci across their length. Eyes prominent. Antennomere 2 shorter than antennomere 3; antennomeres 4–10 about 1.9 times longer than wide.

Mentum with median carina extended distad slightly beyond anterior margin of lateral lobes; pit organs opened through oval orifices to the mental- submental suture.

Pronotum ovate, lateral border reaching base.

Elytra oval, humeri curved, striae complete in their length, impunctate; visible humeral tooth at junction of third stria with marginal channel.

Metathoracic wingsmacropterous.

Genitalia as described for the *puncticollis* species–group.

##### Note.

This species exhibits many plesiotypic character states for the *puncticollis* species-group.

##### Geographical distribution

(Fig. 57)**.** Widespread in the northern parts of South America, east of the Andes mountain range.

##### Habitat and activity.

Records from labels suggest that the habitat of this species is typical of most species of the genus, living on loose soil adjacent to fresh water bodies. Furthermore the species seems to prefer sandy areas with leaves or stones. Tenerals were collected in August.

##### Material examined.

In addition to the type material, I have seen 8 specimens from the following localities.ECUADOR. Pichincha Alturiquin Rio Toachi 9–1 Feb 1999 (PBPC, 2). PERU. Loreto. Boca del Rio Samiria 16 Aug 1991 (USNM, 1); Loreto, Hamburgo, Boca del Inglés 150m 23 Aug 1991 (USNM, 1); Loreto, Pithecia 14 Aug 1989 (USNM, 2); Loreto Cmp. Terry 14 May 1990 (USNM, 1). TRINIDAD AND TOBAGO: Trinidad Chatham 17 Jun 1980 (NMNH, 1)

#### Semiardistomis glabratus

(Putzeys, 1866)

http://species-id.net/wiki/Semiardistomis_glabratus

Figs 3 13 24 26 27 41–43 57

Ardistomis glabrata Putzeys, 1866: 213.
Ardistomus glabratus Putzeys: Csiki 1927: 548; Blackwelder 1944: 27.
Ardistomis glabratus Putzeys: Lorenz 2005: 146.
Ardistomis (Semiardistomis) glabratus Putzeys: Kult 1950: 310.
Ardistomis (Semiardistomis) balthasari Kult, 1950: 309. syn. n.
Semiardistomis glabratus (Putzeys); Erwin 2011: 235.

##### Type material.

Lectotype, here designated, female (IRSNB), glued on cardboard pinned, genitalia attached in microvial, labeled: green paper handwritten "A. glabrata Pz. C Mvideo. (A. 21ª)"/ printed "Soc. Ent. Belg. Coll. Putzeys"/ printed "Syntype."

Paralectotypes, here designated:onefemale (IRSNB) labeled as Lectotype; one exemplar (MHNP) labeled: handwritten in box “glabrata Putz. Mvideo”.

Holotype of *Semiardistomis balthasari* male (ADVA), glued on cardboard pinned, genitalia attached in microvial, labeled: handwritten “Ardistomis balthasari Kt. Det. K. Kult 1948”/ “balthasari Kt. det. K.Kult”/ printed in red paper “TYPE”/ printed “San Ignacio Missions”.

##### Type locality.

Given by Putzeys (1866: 213) as “Montevideo”, Uruguay.

##### Diagnosis.

Body piceous with green brassy reflections. Elytral surface smooth, striae reduced to basal third, impunctate, 4 setiferous punctures on interval 3, stria 2 absent. Profemur glabrous. Abdominal sternum VII with 5+5 setiferous punctures.

##### Habitus.

**Figures 41–44.**
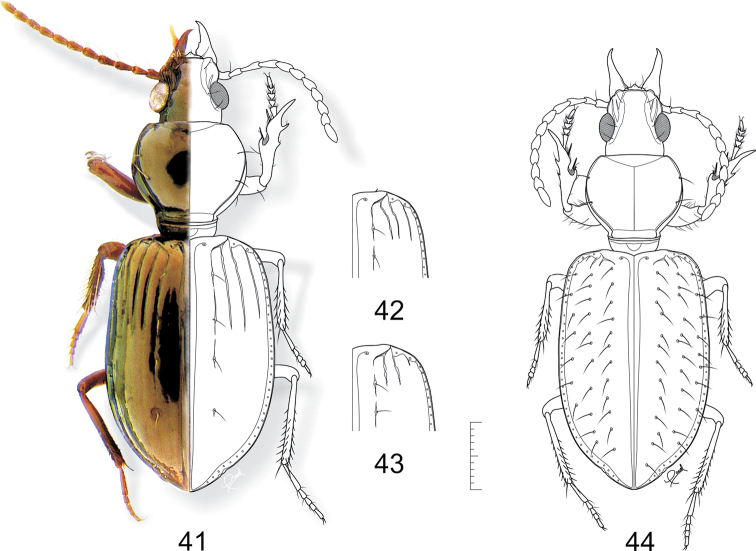
**41–43** *Semiardistomis glabratus* (Putzeys) **41**exemplar from Peru, Loreto **42** exemplar from Argentina, Misiones **43** exemplar from Uruguay, Montevideo **44** *Semiardistomis pilosellus* (Kult). Dorsal aspect. Scale bar 1 mm.

dorsal aspect, as in Fig. 41.

##### Mouthparts.

**Labrum**, dorsal aspect, as in Fig. 3. **Labium**, as in Fig. 13A; ligula, enlarged, as in Fig. 13B.

##### Legs.

male protarsus as in Fig. 24.

##### Male genitalia.

as in Fig. 26.

##### Female genitalia.

as in Fig. 27.

##### Measurements and variation.

Variation of measurements (mm) and ratios for *Semiardistomis glabratus* (n=7) are: HL = 0,70–0,76–0,80; PL 1,44–1,51–1,60; PW 1,53–1,68–1,83; EL 3,56–3,84–4,13; EW 2,50–2,60–2,73; SBL 5,76–6,11–6,53; PW/EW 0,65; PW/PL 1,11; PL/EL 0,39; EW/EL 0,68.

Variation of measurements (mm) and ratios for *Semiardistomis glabratus* identified in collections as *Semiardistomis balthasari* (n= 11) are: HL = 0,67–0,78–0,86; PL 1,12–1,32–1,44; PW 1,34–1,49–1,63; EL 3,33–3,66–3,87; EW 2,08–2,36–2,56; SBL 5,15–5,76–6,11; PW/EW 0,63; PW/PL 1,13; PL/EL 0,36; EW/EL 0,65.

Kult (1950) separated *Semiardistomis balthasari* from *Semiardistomis glabratus* on the basis of two character states: differences in body size and length of elytral striae. Measurements show moderate variation in size between the populations studied (see above) but the ratios show no variations in body proportions. After studying different populations across South America, I found that the length of the elytral striae varies between populations from specimens with striae 4, 5 and 6 extended to the second dorsal puncture in interval 3 (Fig. 42) to specimens with striae 4 and 5 almost extended only to the first dorsal pore in interval 3, and stria 6 almost obliterated (Fig. 43). The pattern of variation is clinal from north to south.

##### Habitat.

Data from labels suggest that the species lives along standing water bodies and swamps.

##### Geographical distribution

(Fig. 57**).** Widespread in South America, east of the Andes mountain range, from northern Brazil, north of the Equator, to Uruguay, south of the Tropic of Capricorn.

##### Material examined.

ARGENTINA. San Ignacio Misiones (ADVA, 2) Rio Salado (IRSNB, 12) Cordoba Argüello Sept 58 (MHNP, 8). BRAZIL. Oberaba (IRSNB, 9). PERU. Loreto 1 km E Hamburgo, Boca del Ingles Camp, 150 m 23 Aug 1991 (USNM, 8). Loreto, Pithecia 14 Aug 1989 (USNM, 5). Madre de Dios Pakitza 07 Oct 1990 (USNM, 5). URUGUAY. Montevideo (IRSBN, 5) (MHNP, 2). VENEZUELA. Amazonas, Dpto Rio Negro 28 Jan 1985 (USNM, 4).

#### Semiardistomis jedlickai

(Kult, 1950)

http://species-id.net/wiki/Semiardistomis_jedlickai

Figs 45 57

Ardistomis (Semiardistomis) jedlickai Kult, 1950: 313.
Semiardistomis jedlickai (Kult): Lorenz 1998: 136.

##### Type material.

Holotype at ADVA, glued on pinned card (specimen without genitalia), labeled: handwritten “Ardistomis jedlickai Kt. Det. K. Kult 1948”/ “jedlickai Kt. det. K.Kult”/ printed in red paper “TYPE”/ printed “Corumba Matt. Grosso.”

##### Type locality.

Given by Kult (1950: 313) as “Brasilia, Matto Grosso, Corumba”, Brazil.

##### Diagnosis.

Body piceous with green brassy reflections. Elytral surface smooth, striae distinct in basal half only, impunctate, 4 setiferous punctures on interval three, stria 2 distinct. Profemur glabrous. Abdominal sternum VII with 4+4 setiferous punctures. Size small.

##### Habitus.

**Figures 45–46.**
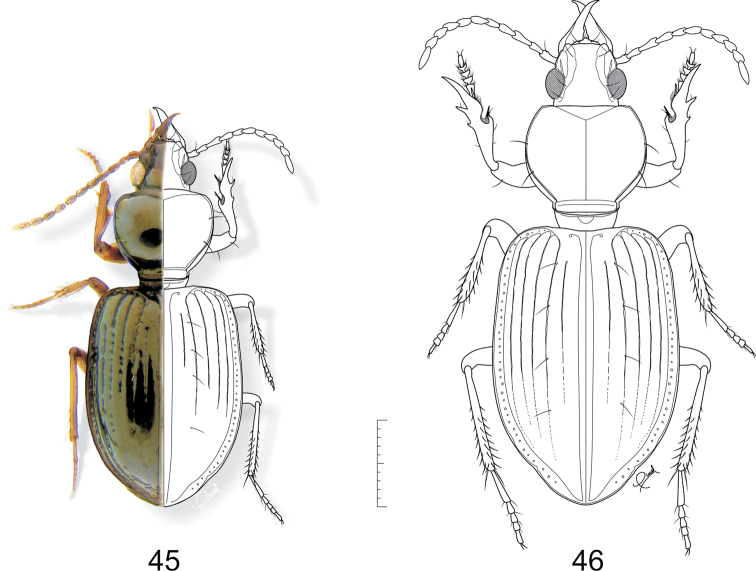
**45** *Semiardistomis jedlickai* (Kult) **46** *Semiardistomis maindroni* (Kult). Dorsal aspect. Scale bar 1 mm.

dorsal aspect, as in Fig. 45.

##### Measurements and variation.

Variation of measurements (mm) and ratios for *Semiardistomis jedlickai* (n=6) are: HL = 0,50–0,51–0,53; PL 0,96–1,00–1,07; PW 0,99–1,17–1,28; EL 2,30–2,55–2,62; EW 1,70–1,82–1,92; **SBL** 3,82–4,07–4,19; PW/EW 0,64; PW/PL 1,16; PL/EL 0,39; EW/EL 0,71.

##### Geographical distribution

(Fig. 57)**.** The known range of this species is confined to central South America.

##### Material examined.

In addition to holotype: BRAZIL. Matto Grosso Jacare P. N. Xingu at ligth (USNM, 11) PERU. Madre de Dios Pakitza 14 Nov 1990 (USNM, 6). Madre de Dios Rio Manu BIOLAT Pakitza 356m 24 Jun 1993 (USNM, 5).

#### Semiardistomis laevistriatus

(Fleutiaux & Sallé, 1889)

http://species-id.net/wiki/Semiardistomis_laevistriatus

Figs 47 57

Ardistomis laevistriatus Fleutiaux & Sallé, 1889: 363; Leng and Mutchler 1914: 395; Kult 1950: 307; Erwin and Sims 1984: 427; Lorenz 2005: 146.
Ardistomus laevistriatus Fleutiaux & Sallé: Csiki 1927: 548; Blackwelder 1944: 27.
Semiardistomis laevistriatus (Fleutiaux & Sallé); Erwin 2011: 236.

##### Type material.

Lectotype, here designated, male (MHNP), glued on pinned card, labeled: printed “Type”; printed “Guadeloupe Delauney”; handwritten “Ardistomis laevistriata Fleut. & S. type”; “Ardistomis laevistriata Fleut. 47 det. K. Kult type”; “Lectotype Ardistomis laevistriata F. & S. des. S. W. Nichols 1984.”

Paralectotype, here designated,female (MHNP), glued on pinned card, genitalia attached in microvial, labeled: handwritten on paper circle "Guadeloupe Delauney"; printed "ex Musaeo A. Sallé 1897"; "Paralectotype Ardistomis laevistriata F. & S. des. S. W. Nichols 1984”.

##### Type locality.

Given by Fleutiaux and Sallé (1889: 364) as “Les Bains- Jaunes”, Guadeloupe, Lesser Antilles.

##### Diagnosis.

Body ferrugineous dark brown. Elytral surface smooth, striae obliterated in apical fifth, impunctate, 2 setiferous punctures on interval 3, stria 2 distinct, shoulders rounded. Metathoracic wings reduced. Profemur glabrous. Abdominal sternum VII with 5+5 setiferous punctures.

##### Habitus.

**Figures 47–48.**
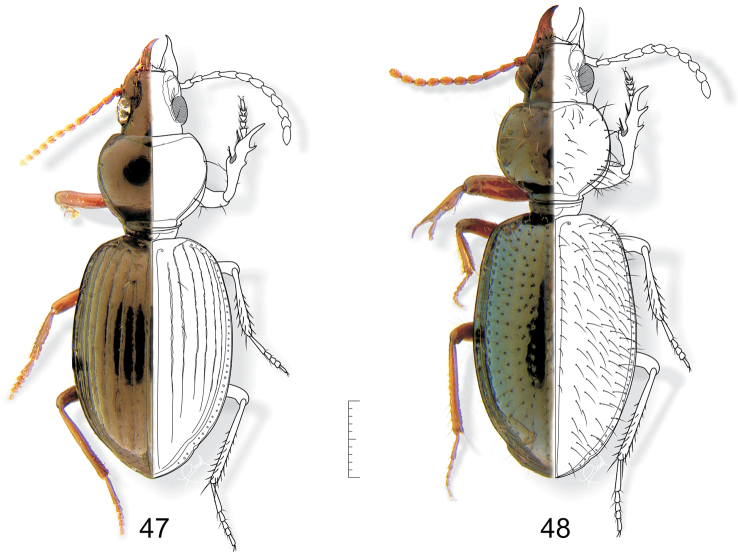
**47** *Semiardistomis laevistriatus* (Fleutiaux & Salle) **48** *Semiardistomis puncticollis* (Dejean). Dorsal aspect. Scale bar 1 mm.

dorsal aspect, as in Fig. 47.

##### Measurements and variation.

Variation of measurements (mm) and ratios for *Semiardistomis laevistriatus* (n=4) are: HL = 0,65–0,68–0,70; PL = 1,13–1,20–1,28; PW = 1,33–1,39–1,45; EL = 2,95–3,05–3,15; EW = 2,00–2,05–2,10; **SBL =** 4,73–4,93–5,13; PW/EW = 0,68; PW/PL = 1,16; PL/EL = 0,39; EW/EL = 0,67.

##### Habitat.

Nichols (1988a) reported that adults of *Semiardistomis laevistriatus* are found under rotting bark. The species is probably associated with wet forest leaf litter.

##### Geographical distribution

**(Fig. 57).** Restricted to the Islands of Guadeloupe in the Lesser Antilles.

##### Material examined.

GUADELOUPE. Guadeloupe L. Dufau (ADVA, 1) Bains–Jaunes (USNM, 2). Guadeloupe Marie (MHNP, 1).

#### Semiardistomis maindroni

(Kult, 1950)

http://species-id.net/wiki/Semiardistomis_maindroni

Figs 46 57

Ardistomis (Semiardistomis) maindroni Kult, 1950: 312.
Semiardistomis maindroni (Kult, 1950): Lorenz 1998: 136.

##### Type material.

Holotype at ADVA, glued on cardboard pinned, genitalia attached in microvial, labeled: handwritten “Ardistomis maindroni Kt. Det. K. Kult 1948”/ “maindroni Kt. det. K.Kult”/ printed in red paper “TYPE”/ printed “Cuyaba Matt. Grosso”.

Paratypes. two exemplars labeled as holotype (not checked) designated by Kult in “collection of National Museum of Paris (ex Maindron- Babault)”.

##### Type locality.

Given by Kult (1950: 312) as “Brasilia, Matto Grosso, Cuyaba”, Brazil.

##### Diagnosis.

Body piceous with green reflections. Elytral surface covered with isodiametric mesh pattern, striae obliterated in apical half, impunctate, 4 setiferous punctures on interval 3, stria 2 present. Profemur glabrous. Abdominal sternum VII with 4+4 setiferous punctures.

##### Habitus.

dorsal aspect, as in Fig. 46.

##### Measurements and variation.

Variation of measurements (mm) and ratios for *Semiardistomis maindroni* (n=9) are: HL = 0,63–0,66–0,71; PL = 1,18–1,25–1,32; PW = 1,31–1,44–1,57; EL = 3,10–3,38–3,64; EW = 2,02–2,22–2,40; **SBL =** 4,93–5,30–5,60; PW/EW = 0,65; PW/PL = 1,15; PL/EL = 0,37; EW/EL = 0,67.

##### Geographical distribution

**(Fig. 57).** The known range of this species extends from central Brazil southward of the Tropic of Capricorn to central Argentina.

##### Material examined.

ARGENTINA. Cordoba Arguello Nov 1958 (MHNP, 5); Tucuman Nov 1951 (MHNP, 6); Chaco prov. Capitán Solari env. 3–7 Feb. 2004 (PBPC, 2). BRAZIL. Porta da Bocca am Lagua de Jigua Pernambuco Jul 1937 (BMNH, 4).

#### Semiardistomis major sp. n.

urn:lsid:zoobank.org:act:86C18C17-48F7-404A-B43B-76F1707EE250

http://species-id.net/wiki/Semiardistomis_major

Figs 49 57

##### Holotype.

Male (USNM), glued on pinned point, genitalia attached in microvial, labeled: printed "Peru: Loreto, Cmp. S. Branch, 11 May 90 75°20'W, 05°12'S T. L. Erwin Coll"/ "Running at night on muddy bank of Rio Samiria–Igapó forest soil organic, grasses"/ "ADP 94456".

##### Paratypes.

8 exemplars (USNM), 2 exemplars (PVCCu) labeled as the holotype.

##### Etymology.

The specific epithet is a Latin adjective alluding to the large size of the adults.

##### Diagnosis.

Elytral surface smooth, striae complete, impunctate, 4 setiferous punctures on interval 3, shoulders rounded. Profemur glabrous. Abdominal sternum VII with 5+5 setiferous punctures.

##### Habitus.

**Figures 49–50:**
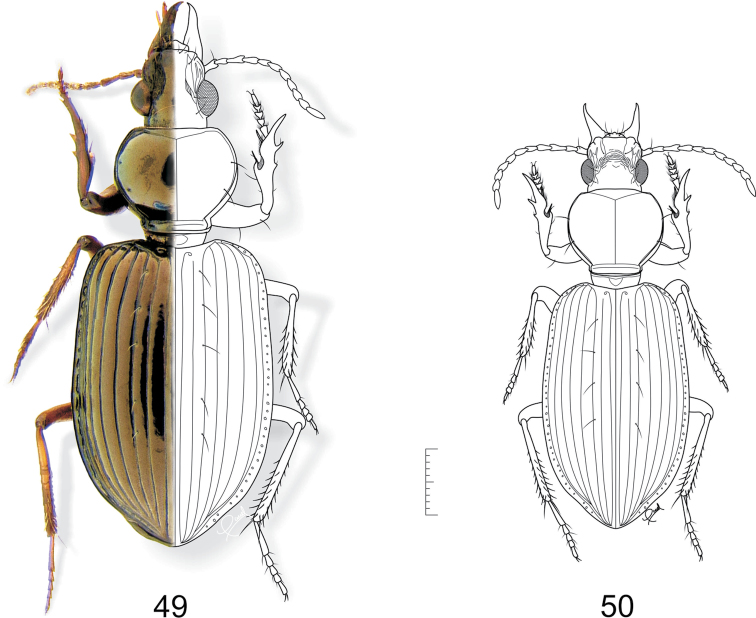
**. 47** *Semiardistomis major* sp.n. **50** *Semiardistomis rugosus* (Putzeys). Dorsal aspect. Scale bar 1 mm.

dorsal aspect, as in Fig. 49.

##### Measurements and variation.

Variation of measurements (mm) and ratios for *Semiardistomis major* **sp. n**. (n=11) are: HL = 0,93–0,97–0,99; PL = 1,50–1,61–1,66; PW = 1,92–1,98–2,02; EL = 4,47–4,71–4,80; EW = 3,13–3,23–3,33; **SBL =** 6,90–7,28–7,46; PW/EW = 0,61; PW/PL = 1,23; PL/EL = 0,34; EW/EL = 0,69.

##### Description.

Body piceous with green reflections, mouthparts and antennae testaceous, legs ferrugineous.

Frons, gena, antennal lobes, pronotum, prosternum, proepisternum, elytra, metasternum and abdominal segments smooth.

Anterior marginal setae on pronotal disc closer to posterior setae than to anterior angles. Elytral disc with 5 setae in interval 3. Abdominal sternum VII with setae near base, 2 on each side; distance between inner pair of preapical setae 0.6 times that between inner and outer setae. Profemur glabrous. Ventral surface of protibia with many setae on basal half.

Clypeus with anterior margin concave medially; lateral lobes distinct, behind level of anterior margin. Frontal impressions deep and wide. Supraantennal lobes with median sulci impressed in basal half. Eyes prominent. Antennomere 2 shorter than antennomere 3; antennomeres 4–10 about 1.8 times longer than wide.

Mentum with median carina extended distad slightly beyond anterior margin of lateral lobes; pit organs opened through oval orifices to the mental- submental suture.

Pronotum ovate, lateral border extended to base.

Elytra oblong, humeri square, striae complete in their length, impunctate.

Metathoracic wingsmacropterous.

Genitalia in both sexes as described for the *puncticollis* species–group.

##### Geographical distribution

(Fig. 57)**.** Known only from the region of Loreto, in Amazonian Peru.

##### Habitat and activity.

Records from labels suggest that the habitat of *Semiardistomis major* is typical of most members of the genus, living on loose soil adjacent to fresh water bodies. In this case data indicate a river bank with organic soil. Active at night.

##### Material examined.

PERU. Loreto. Rio Samiria Cocha Shinguito near lake margin 25 Aug 1991 (USNM, 1).

#### Semiardistomis pilosellus

(Kult, 1950)

http://species-id.net/wiki/Semiardistomis_pilosellus

Figs 44 57

Ardistomis (Semiardistomis) pilosellus Kult, 1950: 317.
Semiardistomis pilosellus (Kult): Lorenz 1998: 136.

##### Type material.

Holotype female (IRSNB), pinned, genitalia attached in microvial, labeled: green paper handwritten “pilosella. P.”/ green paper handwritten “Mvid Arech.”/ handwritten “Ardistomis pilosella 47 det. K. Kult”/ printed “Soc. Ent. Belg. Coll. Putzeys” red paper printed “TYPE”/ printed “Holotype Ardistomis pilosella Kult des. S. W. Nichols 1984”

Paratypes. Male (ADVA), pinned, genitalia attached in microvial, labeled: handwritten “Montevideo leg. Arechavaleta”/ green paper handwritten “Mvid Arech.”/ handwritten “Ardistomis pilosella 47 det. K. Kult”/ handwritten “pilosellus Kt det. K. Kult”/ printed “Soc. Ent. Belg. Coll. Putzeys”/ red paper printed “Paratype”.

##### Type locality.

Given by Kult (1950: 317) as “Uruguay, Montevideo”

##### Diagnosis.

Body piceous with green brassy reflections. Elytral surface smooth, striae 2–6 erased, surface covered with setiferous punctures. Profemur setose. Abdominal sternum VII with 5+5 setiferous punctures.

##### Habitus.

dorsal aspect, as in Fig. 44.

##### Measurements and variation.

Variation of measurements (mm) and ratios for *Semiardistomis pilosellus* (n=6) are: HL = 0,70–0.75–0.80; PL = 1,16–1,23–1,31; PW = 1,30–1,35–1,44; EL = 3,40–3,50–3,64; EW = 2,12–2,19–2,27; **SBL =** 5,26–5,48–5,70; PW/EW = 0,62; PW/PL = 1,17; PL/EL = 0,37; EW/EL = 0,64.

##### Geographical distribution

(Fig. 57)**.** This species is found south of the Rio de la Plata–Uruguay basin, south of the Tropic of Capricorn.

##### Material examined.

ARGENTINA. Pronunciamiento Prov. Entre Rios (MHNP, 12).

#### Semiardistomis puncticollis

(Dejean, 1831)

http://species-id.net/wiki/Semiardistomis_puncticollis

Figs 48 57

Clivina puncticollis Dejean, 1831: 508; Lectotype designated by Bousquet (2006: 14).
Ardistomis puncticollis (Dejean): Putzeys 1846: 647; LeConte 1857: 80; LeConte 1879: 32; Blatchley 1910: 63; Downie and Arnett 1996: 112.
Ardistomus puncticollis (Dejean): Csiki 1927: 548.
Semiardistomis puncticollis (Dejean); Erwin 2011: 238.

##### Type material.

Lectotype at (MHNP), glued on tip pinned, labeled: printed “Lectotype”; handwritten “36”; “puncticollis Dej. Am. bor. Dej.”; “Lectotype Clivina puncticollis Dej. des. S. W. Nichols 1984.”.

##### Type area.

Given by Dejean (1831: 509) as "Amérique septentrionale."

##### Diagnosis.

Body piceous with green reflections. Head with several supraorbital setae. Pronotum with more than two premedial setiferous punctures, pronotal disc with several setiferous punctures. Elytral surface completely smooth, striae absent, punctures deeply impressed, setiferous. Abdominal sternum VII plurisetose. Wing atrophy reported by Nichols (1998a: 193). Body length given by Bousquet (2006: 14) as 4.8–6.5 mm.

##### Habitus.

dorsal aspect, as in Fig. 48.

##### Geographical distribution

(Fig. 57)**.** Known only from southeastern United States and adjacent northeastern Mexico (Nichols 1988a; Bousquet 2006).

##### Material examined. 

USA. Enterprise, Fla 13 5 (USNM, 2) Crescent City, Fla (USNM, 1). L. Harney, Fla. May (USNM, 2) San Jacinto Co. Tex, Jun 68 (USNM, 3) Etats unis Guer (MHNP, 5).

#### Semiardistomis rugosus

(Putzeys, 1866)

http://species-id.net/wiki/Semiardistomis_rugosus

Figs 50 57

Ardistomis rugosa Putzeys, 1866: 210.
Ardistomus rugosus Putzeys: Csiki 1927: 549; Blackwelder 1944: 27.
Ardistomis (Semiardistomis) rugosus Putzeys: Kult 1950: 302; Lorenz 2005: 146.
Semiardistomis rugosus (Putzeys); Erwin 2011: 238.

##### Type material.

Lectotype, here designated, female (IRSNB), glued on pinned card, head and pronotum missing, genitalia attached in microvial, labeled: green paper handwritten “rugosa. Pz. Sta Cath.(Dhr.)”/ printed “Soc. Ent. Belg. Coll. Putzeys”/ “Syntype”/ “Lectotype Ardistomis rugosa Putz. des. S. W. Nichols 1984”.

Putzeys (1866: 211) mentioned two specimens in his description but I have located only one.

##### Type locality.

Given by Putzeys (1866: 211) as “Ste Catherine (Colonie Blumenau)”, Brazil.

##### Diagnosis.

Body piceous. Elytral surface smooth, impunctate, elytral striae complete, 4 setiferous punctures on interval 3, stria 2 absent, prominent humeral tooth at junction of third stria with marginal channel. Profemur glabrous. Abdominal sternum VII with 5+5 setiferous punctures.

##### Habitus.

dorsal aspect, as in Fig. 50.

##### Measurements and variation.

Variation of measurements (mm) and ratios for *Semiardistomis rugosus* (n=2) are: HL = 0,71; PL = 1,35; PW = 1,51; EL = 3,72–3,73–3,75; EW = 2,34–2,42–2,50; **SBL =** 5,78; PW/EW = 0,64; PW/PL = 1,12; PL/EL = 0,36; EW/EL = 0,63.

##### Geographical distribution

(Fig. 57)**.** Known only for the Santa Catarina region in Brazil.

##### Material examined.

BRAZIL. Hansa Humboldt Sta. Catarina 3. 1937 (BMNH, 1).

#### Semiardistomis subglabra

(van Emden, 1949)

http://species-id.net/wiki/Semiardistomis_subglabra

Figs 40 57

Ardistomis subglabra van Emden, 1949: 863; Lorenz 2005: 146.
Ardistomis (Semiardistomis) vlastae Kult, 1950: 310 syn. n.
Semiardistomis subglabra (van Emden); Erwin 2011: 239.

##### Type material.

Holotype female (BMNH), glued on pinned point, without head, genitalia attached in microvial, labeled: green paper handwritten “subglabra Emd.”/ red paper handwritten “Ardistomis subglabra type. Emd.”/ green paper handwritten “amazon”/ printed red circle “Holotype”/ printed “Gesch. 2. 1934 von Prf. Noesske”/ printed “F. van Emden Bequest. B.M. 1960- 129”/ printed “Holotype Ardistomis subglabra Emden des. S.W. Nichols 1984”.

Holotype of *Semiardistomis vlastae* male (ADVA), glued on pinned card, genitalia attached in microvial, labeled: handwritten “vlastae Kt. det. K.Kult”/ printed in red paper “TYPE”/ printed “Chaco Pilcomayo 5-6 Jan. 1938 Tippmann”.

##### Type area.

Given by van Emden (1949: 863) as “Amazonas”, Brazil.

##### Diagnosis.

Body piceous with green bluish reflections. Elytral surface smooth, impunctate, striae distinct on basal third only, impunctate, 8 setiferous punctures on interval 3, stria 2 absent. Profemur hirsute. Abdominal sternum VII with 6+6 setiferous punctures.

##### Habitus.

dorsal aspect, as in Fig. 40.

##### Measurements and variation.

Variation of measurements (mm) and ratios for *Semiardistomis subglabra* (n=4) are: HL = 0,60–0,70–0,78; PL = 1,12–1,21–1,28; PW = 1,23–1,34–1,44; EL = 3,50–3,64–4,75; EW = 2,05–2,25–2,40; **SBL =** 5,28–5,52–5,78; PW/EW = 0,60; PW/PL = 1,11; PL/EL = 0,33; EW/EL = 0,62.

##### Geographical distribution

**(Fig. 57).** Widespread in South America.

##### Material examined.

ARGENTINA. Chaco Pilcomayo 5-6 Jan 1938 (ADVA, 1). BRAZIL. Pantanal Jun 1986 (PBPC, 2).

## The *labialis* species–group

This group is defined by the following: mentum with median carina (Fig. 14C) not extended distad, beyond anterior margin of mental tooth; mental pit organs (Fig. 14A) opened through oval orifices in basal part of mentum; male genitalia with a slender phallus (Fig. 25A) with clearly delineated basal bulb (**bp**) and basal sclerite (**bs**) of the endophallus thin and slightly sclerotized (Fig. 25B); female reproductive tract (Fig. 28; cf. Fig. 27) with spermatheca duct (**spd**) relatively long, wide, bent in its distal portion, spermatheca very narrow, elongate, markedly convoluted in a series of tight twists. The species–group name is based on that of *Semiardistomis labialis*, the type species of *Semiardistomis*, being this group nominotypical.

### Morphological note

As in the *puncticollis* group, the mental carina shows a reduction pattern northward with this prolongation markedly reduced in *Semiardistomis viridis*.

### Included species

*Semiardistomis cyaneolimbatus* (Chevrolat, 1863)

*Semiardistomis deletus* (Putzeys, 1846)

*Semiardistomis flavipes* (Dejean, 1831)

*Semiardistomis labialis* (Chaudoir, 1837)

*Semiardistomis pallipes* (Dejean, 1831)

*Semiardistomis propinquus* (Putzeys, 1866)

*Semiardistomis semipunctatus* (Dejean, 1831)

*Semiardistomis viridis* (Say, 1823)

#### Semiardistomis cyaneolimbatus

(Chevrolat, 1863)

http://species-id.net/wiki/Semiardistomis_cyaneolimbatus

Figs 51 53 59

Ardistomis cyaneolimbatus Chevrolat, 1863: 194; Putzeys 1866: 211; Leng and Mutchler 1914: 395; Darlington 1934: 71; Erwin and Sims 1984: 427; Lorenz 2005: 146.
Ardistomus cyaneolimbatus Chevrolat: Csiki 1927: 548; Blackwelder 1944: 27.
Ardistomis (Semiardistomis) cyaneolimbatus Chevrolat: Kult 1950: 300, 316.
Ardistomis gundlachii Putzeys, 1866: 212; Gundlach 1891: 25. *Nomen nudum*.
Semiardistomis cyaneolimbatus (Chevrolat); Erwin 2011: 233.

##### Type material.

Lectotype, here designated, male (HECO), pinned, labeled: green paper handwritten “Cuba Poey”/ handwritten “ Aspidoglossa cyaneolimbata Chevt. Type Cuba 1461.”/ printed “Chevrolat Carabidae. Fr. V. d. Poll. Pres. 1909, E. B. Poulton”/ printed “type col: 132 Ardistomus cyaneolimbata Chevr. Hope Dept. Oxford”/ printed “Lectotype Ardistomis cyaneolimbatus Chev. des. S.W. Nichols 1984”..

##### Type locality.

Given by Chevrolat (1863: 194) as “environs de la Havane”, Cuba.

##### Diagnosis.

Body piceous with green brassy reflections, pronotum with bluish reflections. Elytral surface smooth, striae complete but indistinct at apex, punctures moderately impressed, 3 setiferous punctures on interval 3. Abdominal sternum VII with 4+4 setiferous punctures.

##### Habitus.

**Figures 51–52.**
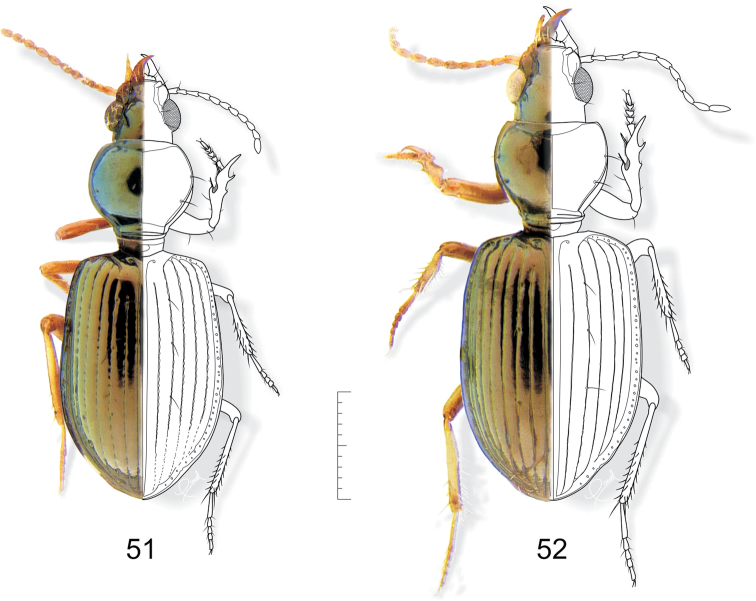
**51** *Semiardistomis cyaneolimbatus* (Chevrolat) **52** *Semiardistomis pallipes* (Dejean). Dorsal aspect. Scale bar 1 mm.

dorsal aspect, as in Fig. 51.

##### Measurements and variation.

Variation of measurements (mm) and ratios for *Semiardistomis cyaneolimbatus* (n=7) are: HL = 0,52- 0,59- 0,60; PL = 1,00- 1,03- 1,10; PW = 1,10- 1,18- 1,30; EL = 2,44- 2,72- 2,84; EW = 1,60- 1,75- 1,88; **SBL =** 3,96- 4,34- 4,54; PW/EW = 0,67; PW/PL = 1,14; PL/EL = 0,38; EW/EL = 0,64.

##### Geographical distribution

(Fig. 59)**:** West Indies: Cuba, Cayman Islands and Haiti, in Hispaniola.

##### Habitat and activity.

**Figure 53.**
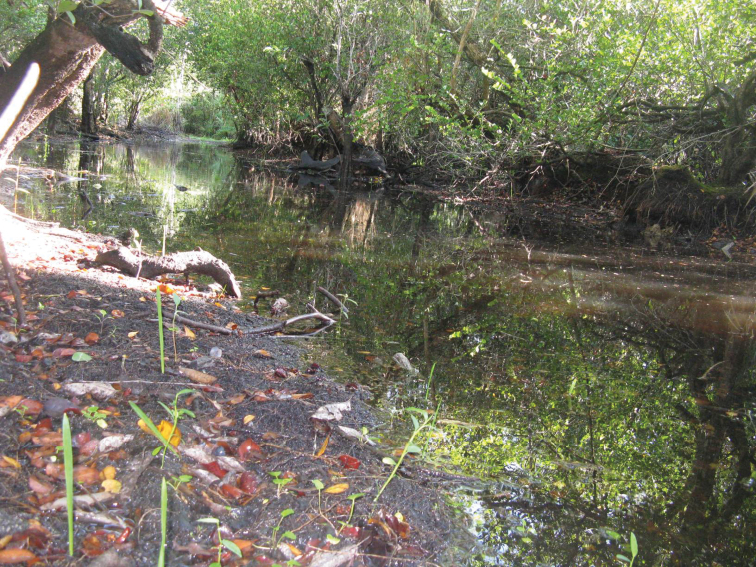
Habitat of *Semiardistomis cyaneolimbatus* (Chevrolat) at Rio Itabo, Isla de Pinos, Cuba.

I collected this species along margins of the river Itabo, Isla de Pinos, Cuba (Fig. 53); the adults were active during the day on wet, organic soil. Other specimens were collected about 8 meters from the river margins under leaf litter in shady gallery forest.

##### Material examined.

CUBA. Cuba Rhl (IRSNB, 4); Cuba Chd (MHNP, 1); gundlachii Ptz. Cuba Type (MHNP, 1); Isla de Pinos, márgenes del Rio Itabo 23 Feb. 1999 (PVCCu: 14). HAITI. St. Dominique (MHNP, 1).

#### Semiardistomis deletus

(Putzeys, 1846)

http://species-id.net/wiki/Semiardistomis_deletus

Figure 58

Ardistomis deleta Putzeys, 1846: 648; 1866: 213.
Ardistomus deletus Putzeys: Csiki 1927: 548; Blackwelder 1944: 27.
Ardistomis deletus Putzeys: Lorenz 2005: 146.
Ardistomis (Semiardistomis) deletus Putzeys: Kult, 1950: 314, 316.
Ardistomis (Semiardistomis) emdeni Kult, 1950: 314, syn. n.
Semiardistomis deletus (Putzeys); Erwin 2011: 234.

##### Type material.

Lectotype, here designated,at MHNP, glued on cardboard, labeled: green paper handwritten "Deleta Putz Bresil Reiche"/ handwritten " Lectotype Ardistomis deleta Putz. des. S.W. Nichols 1984"/ handwritten on box "Deleta Reiche Brésil Chevrol"/ red paper printed "Lectotype".

Paralectotype, here designated, at HECO, pinned, labeled: green paper handwritten “Campos”/ green paper handwritten “deleta (Reiche) Putz 130, 13 Brasilia type”/ red paper handwritten “deleta”/ printed “Chevrolat Carabidae. Fr. V. d. Poll. Pres. 1909, E. B. Poulton”/ printed “17”/ handwritten “Paralectotype Ardistomis deleta Putz. des. S.W. Nichols 1984”/ handwritten “Type col: 805 Ardistomus deleta Putzeys”.

Holotype of *Semiardistomis emdeni* female (ADVA), glued on pinned card, genitalia attached in microvial, labeled: handwritten “a. emdeni Kt. det. K.Kult, 1948”/ printed on red paper “TYPE”/ printed “Corumba Matt. Grosso”.

##### Type area.

Given by Putzeys (1846: 648) as "Brésil"

##### Diagnosis.

Body piceous with green brassy reflections. Elytral surface with isodiametric mesh pattern, striae impressed in basal eighth, punctures well impressed in basal half, 3 setiferous punctures on interval 3. Abdominal sternum VII with 4+4 setiferous punctures.

##### Measurements and variation.

Variation of measurements (mm) and ratios for *Semiardistomis deletus* (n=17) are: HL = 0,53- 0,63- 0,74; PL = 0,80- 1,06- 1,20; PW = 0,96- 1,19- 1,32; EL = 2,56- 3,11- 3,60; EW = 1,60- 1,93- 2,28; **SBL =** 3,98- 4,80- 5,44; PW/EW = 0,62; PW/PL = 1,12; PL/EL = 0,34; EW/EL = 0,62.

Variation of measurements (mm) and ratios for type series of *Semiardistomis emdeni* (n=5) are: HL = 0,58–0,61–0,64; PL = 1,02–1,05–1,09; PW = 1,12–1,20–1,28; EL = 2,78–2,95–3,14; EW = 1,76–1,89–2,02; **SBL =** 4,38–4,61–4,86; PW/EW = 0,64; PW/PL = 1,15; PL/EL = 0,36; EW/EL = 0,64.

Kult (1950) separated *Semiardistomis emdeni* from *Semiardistomis deletus* based on differences in body proportions. My measurements indicate that the differences noted are not significant. In fact, it is difficult in this species–group to distinguish any forms based on body proportions since measurements for all species overlap. Diagnostic combination for this species is expressed in an extreme sense, so intermediate forms are found between this point and *Semiardistomis flavipes* or *Semiardistomis semipunctatus*.

##### Geographical distribution

(Fig. 58)**.** This species is known from southeastern South America, south of the Tropic of Capricorn.

##### Material examined.

ARGENTINA. Corrientes, Lago Ibera & Santo Tome 26 Sep 1997 (ADVA, 22); Rio Salado (IRSNB, 3); Misiones, Posadas Nov 1962 (MHNP, 6); Formosa Dec 1953 (MHNP, 11). BRAZIL. Nova Teutonia Dec. 1952 (MHNP, 8); Bresil (MHNP, 9); Sta Catarina Nova Teutonia Nov. 1972 (CAS, 17). PARAGUAY. Paraguari Ybycui, La Rosada 13 Apr. 1980 (USNM, 43); Dep. Central. Caacupé road Arroyo Yagua Resa 10 Apr. 1980 (USNM, 2) URUGUAY. Montevideo (IRSBN, 1).

#### Semiardistomis flavipes

(Dejean, 1831)

http://species-id.net/wiki/Semiardistomis_flavipes

Fig. 58

Clivina flavipes Dejean, 1831: 510.
Ardistomis flavipes (Dejean): Putzeys 1846: 646; Lorenz 2005: 146.
Ardistomus flavipes (Dejean): Csiki 1927: 548; Blackwelder 1944: 27.
Ardistomis aenea Putzeys, 1866: 212 syn. n.
Ardistomis (Semiardistomis) britoni Kult, 1950: 316 syn. n.
Ardistomis (Semiardistomis) marani Kult, 1950: 315 syn. n.
Semiardistomis flavipes (Dejean); Erwin 2011: 234.

##### Type material.

Holotypeat MHNP, pinned, labeled: handwritten “ Holotype Clivina flavipes Dej. des. S.W. Nichols 1984”/ handwritten on box “flavipes Dej. Brésil C. Dejean"/ red paper printed "Holotype".

Lectotype of *Semiardistomis aenea*, here designated, at IRSBN, glued on pinned card, labeled: green paper handwritten “R. Jan. Stevens”/ printed “Soc. Ent. Belg. Coll. Putzeys”. Paralectotypes, here designated: 4 at IRSBN labeled as Lectotype. 1 at MHNP labeled as Lectotype.

Holotype of *Semiardistomis brittoni* at ADVA labeled: handwritten “Ardistomis brittoni Kt. Det. K. Kult 1948”/ “brittoni Kt. det. K.Kult”/ printed in red paper “TYPE”/ handwritten “Argentina Tucuman”.

Holotype of *Semiardistomis marani* at ADVA labeled: handwritten “Ardistomis brittoni Kt. Det. K. Kult 1946”/ “brittoni Kt. det. K.Kult”/ printed in red paper “TYPE”/ handwritten “Bolivia 450m Sta Cruz Umg Buenavista Steinbach coll”.

##### Type area.

Given by Dejean (1831: 510) as "Brésil".

##### Diagnosis.

Body piceous with green brassy reflections. Elytral surface with isodiametric mesh pattern, microlines more or less evanescent toward center of elytral disc, striae obliterated in apical fourth, punctures moderately impressed in basal two-thirds, 3 setiferous punctures on interval 3. Abdominal sternum VII with 4+4 setiferous punctures.

##### Measurements and variation.

Variation of measurements (mm) and ratios for *Semiardistomis flavipes* (n=34) are: HL = 0,42–0,59–0,68; PL = 0,96–1,03–1,20; PW = 0,95–1,15–1,34; EL = 2,10–2,86–3,36; EW = 1,44–1,75–2,00; **SBL =** 3,55–4,48–5,16; PW/EW = 0,66; PW/PL = 1,11; PL/EL = 0,36; EW/EL = 0,61.

Variation of measurements (mm) and ratios for *Semiardistomis brittoni* (n=3) are: HL = 0,54–0,62–0,67; PL 0,84–1,03–1,09; PW 1,09–1,21–1,28; EL 2,75–3,03–3,20; EW 1,76–1,90–2,02; **SBL** 4,26–4,68–4,96; PW/EW 0,63; PW/PL 1,16; PL/EL 0,34; EW/EL 0,63.

Variation of measurements (mm) and ratios for *Semiardistomis marani* (n=1) are: HL = 0,58; PL 0,96; PW 1,09; EL 2,62; EW 1,66; **SBL** 4,16; PW/EW 0,65; PW/PL 1,13; PL/EL 0,37; EW/EL 0,63.

Species *Semiardistomis brittoni* and *Semiardistomis marani* measurements are a subset of that of *Semiardistomis flavipes* specimens studied. Variation in this species is found in the microsculpture on the elytral disc which varies from being distinct on the entire surface to being present only marginally, in the extent of the elytral striae, and the size of the punctures. Such variation is seen even within populations from the same locality.

##### Geographical distribution

(Fig. 58)**.** The range of this species extends in central South America from western Brazil southward and westward to Paraguay, south of the Tropic of Capricorn.

##### Habitat and activity.

Label data indicate that specimens of this species were collected during day at the margins of small water bodies, on sand–slit substrate.

##### Material examined.

ARGENTINA. Tucuman (ADVA, 3); Salta Sierra Tartagal 14 Nov. 2006 (ADVA, 6). BOLIVIA. Santa Cruz Buena Vista 20 Feb. 1999 (FSCA, 9) Santa Cruz 450m Buenavista (ADVA, 1). BRAZIL. Mato Grosso, Pantanal 1 Sep. 2000 (PBPC, 2); Rondonia 62 km SW Ariquemes 15- 19 May 1996 (FSCA, 9); Mato Grosso Varzea Grande, Cuiaba 5 May 1972 (FSCA, 10); Minas Geraes (HECO, 3); Rio Jan. (IRSBN, 4). PARAGUAY. Rio Confusa 18 Jan. 1937 (IRSBN, 144)

#### Semiardistomis labialis

(Chaudoir, 1837)

http://species-id.net/wiki/Semiardistomis_labialis

Figs 1, 2 4 6 14 22, 23 25 28 59

Clivina labialis Chaudoir, 1837: 18.
Ardistomis labialis (Chaudoir): Putzeys 1846: 648 ; Bates 1881: 35 ; Lorenz 2005: 146.
Ardistomus labialis (Chaudoir): Csiki 1927: 548; Blackwelder 1944: 27.
Ardistomis (Semiardistomis) labialis (Chaudoir): Kult 1950: 301.
Ardistomis (Semiardistomis) labialis var. picipes Bates, 1881: 35.
Ardistomis (Semiardistomis) labialis var. nanus Bates, 1881: 35.
Ardistomis (Semiardistomis) labialis var. dilatatus Bates, 1881: 35.
Ardistomis tuspanensis Putzeys 1846: 649 syn. n.
Semiardistomis labialis (Chaudoir); Erwin 2011: 235.

##### Type material.

Lectotype, here designated, at MHNP, glued on point, labeled: green paper handwritten “Labialis Chaud Mexico Dej.”/ handwritten in box “labialis Chaud Mexique Chevrol”/ handwritten “38”/ “Lectotype Ardistomis labialis Chd. Det George E. Ball 72”/ “Lectotype Clivina labialis Chd. Des. S.W. Nichols 1984”/ printed in red paper “lectotype”/ printed in circle “Lectotype

Paralectotypes, here designated: two exemplars at MHNP labeled: handwritten in box “labialis Chaud Mexique Chevrol”; handwritten “Paralectotype Clivina labialis Chd. Des. S.W. Nichols 1984”.

Lectotype of *Ardistomis tuspanensis*, here designated, at IRSBN labeled: green paper handwritten “ Tuspanensis Tuspan (Chev.)”/ printed “Soc. Ent. Belg. Coll. Putzeys”/ handwritten “P. Basilewsky Ardistomus tuspanensis Putz”.

##### Type area.

Given by Chaudoir (1837: 19) as “Mexique”.

##### Diagnosis.

Body piceous with green reflections. Elytral surface completely smooth, striae continuously impressed in basal 1/8, punctures deeply impressed in basal 2/3, 3 setiferous pores on interval 3. Abdominal sternum VII with 4+4 setiferous punctures.

##### Habitus.

dorsal and ventral aspects, respectively, as in Figs 1, 2.

##### Mouthparts.

**Labrum**, dorsal aspect, as in Fig. 4. **Labium**, as in Fig. 14A; ligula, enlarged, as in Fig. 14B..

**Legs:** male protarsus as in Fig. 23.

**Male genitalia:** as in Fig. 25.

**Female genitalia:** as in Fig. 28**.**

##### Measurements and variation.

Variation of measurements (mm) and ratios for *Semiardistomis labialis* from Mexico (n=11) are: HL = 0,53–0,58–0,68; PL = 0,90–1,11–1,25; PW = 1,00–1,23–1,40; EL = 2,65–3,10–3,40; EW = 1,70–1,94–2,20; **SBL =** 4,10–4,80–5,23; PW/EW = 0,63; PW/PL = 1,10; PL/EL = 0,36; EW/EL = 0,63.

Variation of measurements (mm) and ratios for *Semiardistomis labialis* from Costa Rica (n=11) are: HL = 0,54–0,60–0,68; PL 1,00–1,07–1,20; PW 1,10–1,21–1,36; EL 2,60–2,86–3,20; EW 1,68–1,86–2,16; **SBL** 4,14–4,54–5,08; PW/EW 0,65; PW/PL 1,13; PL/EL 0,38; EW/EL 0,65.

Variation of measurements (mm) and ratios for *Semiardistomis labialis* identified in collections as *Semiardistomis tuspanensis* (included syntypes) (n= 5) are: HL = 0,55–0,60–0,68; PL 1,03–1,08–1,23; PW 1,08–1,18–1,30; EL 2,65–2,80–3,00; EW 1,70–1,75–1,85; **SBL** 4,33–4,48–4,63; PW/EW 0,67; PW/PL 1,09; PL/EL 0,39; EW/EL 0,63.

Bates (1881) pointed the high variability of *labialis* populations in Central America. The polymorphic condition of characters, such as body measurements and punctuation of elytra striae, have arose different designations that includes Bates’ varieties and *Semiardistomis tuspanensis* (Putzeys). Body sizes show little variation between geographic regions and since for each measured sample group, distance between extremes measurements is high, this character is useless to identify forms proposed. About elytra disc punctured striae, forms varies in low grades without following any clear geographic pattern having striae punctures weekly to strongly produced and striae almost disappeared (except stria number one) to moderately impressed.

##### Geographical distribution

(Fig. 59)**.** The known range of this species extends in Middle America from central Costa Rica northward to northwestern Mexico (slightly north of the Tropic of Cancer).

##### Activity.

Most label data indicate “attracted to lights”. Tenerals were collected in November.

##### Material examined.

MEXICO. Sinaloa, Culiacán Apr 1969 (USNM, 9) Veracruz, Cordoba Nov. 1966 (USNM, 7) Oaxaca Tehuantepec Dec. 1964 (USNM, 1) Chiapas Tapilulas May 1974 (USNM, 9) Colima Volcano (USNM, 5) Jalapa (IRSNB, 2) Sa. De Durango (IRSNB, 4) Guerrero Chiapas Jul 2005 (PBPC, 2). BELIZE. Sibun River at Gracy Rock Jun 1974 (USNM, 7). GUATEMALA. Alta Verapaz May 1973 (USNM, 40) Sacapulas 4500ft Dec. 1947 (USNM, 3). HONDURAS. Dept. Conayagua Rancho Chiquito May 1964 (UASM, 8), Belize district Jun 1968 (USNM, 18), 5 mi E. Choluteca Jul 1965 (USNM, 22), 16 mi W. Sabana Grande Jul 1965 (USNM, 7), Pespire Jul 1965 (USNM, 7), San Marcus Colon Jul. 1965 (USNM, 10). EL SALVADOR. Ch. Del Guayabo May 1975 (USNM, 1). NICARAGUA. Rivas Rio Canas Gordas Jun 1964 (UASM, 18). COSTA RICA. Guanacaste Santa Rosa NP Jun 2004 (ADVA, 34), Guanacaste NP Jun 1990 (USNM, 9), Guanacaste Santa Cruz Est. Bosque Diria 150- 250 m Nov 1998 (USNM, 18), Guanacaste Santa Rosa 300 m Mar 1990 (USNM, 5).

#### Semiardistomis pallipes

(Dejean, 1831)

http://species-id.net/wiki/Semiardistomis_pallipes

Figs 52 58

Clivina pallipes Dejean, 1831: 510.
Ardistomis pallipes (Dejean): Putzeys 1846: 645; Lorenz 2005: 146.
Ardistomus pallipes (Dejean): Csiki 1927: 548; Blackwelder 1944: 27.
Ardistomus pallipes var. caerulea Putzeys, 1846: 646.
Ardistomis striga Putzeys 1866: 211 syn. n.
Semiardistomis pallipes (Dejean); Erwin 2011: 237.

##### Type material.

Lectotype, here designated, at MHNP, pinned, labeled: handwritten in box “pallipes Dej. Colombie C. Gory”/ handwritten “52”/ “Lectotype Clivina pallipes Dej. Des. S.W. Nichols 1984”/ printed in red paper “lectotype”.

Holotype of *Ardistomis striga* at IRSNB, glued on cardboard, genitalia attached in microvial, labeled: green paper handwritten “A. striga My. Panama. (Mky)”/ printed “Soc. Ent. Belg. Coll. Putzeys”/ red paper printed “Type”/ handwritten “P. Basilewsky Ardistomus striga Putz”/ Holotype Ardistomis striga Putzeys det. DRWhitehead”.

##### Type locality.

Given by Dejean (1831: 511) as “from surroundings of Carthagene”, Colombia.

##### Diagnosis.

Body piceous with green brassy reflections. Elytral surface with isodiametric mesh pattern, striae complete, punctures absent, 3 setiferous punctures on interval 3. Abdominal sternum VII with 4+4 setiferous punctures.

##### Habitus.

dorsal aspect, as in Fig. 52.

##### Measurements and variation.

Variation of measurements (mm) and ratios for *Semiardistomis pallipes* (n=12) are: HL = 0,44–0,54–0,66; PL 0,86–1,01–1,20; PW 0,92–1,10–1,34; EL 2,30–2,74–3,24; EW 1,48–1,72–2,00; SBL 3,70–4,30–5,10; PW/EW 0,64; PW/PL 1,09; PL/EL 0,37; EW/EL 0,63. More western specimens have complete elytral striae, whereas in those from the Amazon Basin the striae are shorter (Fig. 58).

##### Geographical distribution

(Fig. 58)**.** The range of this species extends in South America from northern Brazil and eastern Ecuador north to Colombia, and to Panama in Middle America.

##### Habitat.

Label data indicate that specimens of *Semiardistomis* pallipes were collected at margins of water bodies.

##### Material examined.

BRAZIL. Pernambuco 4 Jan 1883 (USNM, 30). COLOMBIA. Colombie (MHNP, 9); San Alberto 10 Nov 1968 (MHNP, 8). ECUADOR. Napo Onkone Gare Camp 10 Sep. 1995 (FSCA, 3). PANAMA. Panama Mky (IRSNB, 2). PERU. Madre de Dios Rio Manu 18 Jul 1992 (USNM, 24). VENEZUELA. Venz. (IRSNB, 1)

#### Semiardistomis propinquus

(Putzeys, 1866)

http://species-id.net/wiki/Semiardistomis_propinquus

Figs 54 59

Ardistomis propinqua Putzeys, 1866: 214; Bates 1881: 35.
Ardistomus propinquus Putzeys: Csiki 1927: 548; Blackwelder 1944: 27.
Ardistomis propinquus Putzeys: Lorenz 2005: 146.
Ardistomis (Semiardistomis) propinquus Putzeys: Kult 1950: 302.
Semiardistomis propinquus (Putzeys); Erwin 2011: 237.

##### Type material.

Lectotype, here designated, male at IRSNB, glued on card, genitalia attached in microvial, labeled: green paper handwritten "A. propinqua Mex. (Sallé) Chd."/ handwritten " Lectotype Ardistomis propinqua Putzeys det. DRWhitehead"/ "Ardistomus propinquus Putz P Basilewsky 1955"/ printed "Soc. Ent. Belg. Coll. Putzeys"/ "Syntype".

Paralectotypes, here designated: two specimens at IRSNB labeled as the lectotype. Another 3 paralectotypes at MHNP labeled: handwritten in box "propinqua Chaud. Mexique Las Peras. Sallé".

**Type area**. Given by Putzeys (1866: 214) as “Mexique (Oaxaca)”.

**Diagnosis.** Body piceous with green reflections. Elytral surface completely smooth, striae continuously impressed in basal 1/8, punctures deeply impressed in basal 2/3, most punctures with setae, 3 setiferous punctures on interval 3. Abdominal sternum VII with 4+4 setiferous punctures.

**Figures 54–55.**
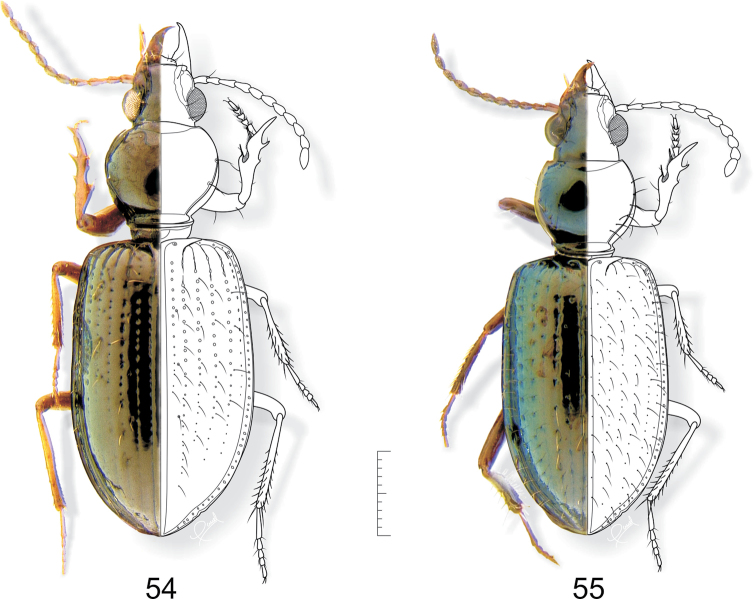
**54** *Semiardistomis propinquus* (Putzeys) **55** *Semiardistomis viridis* (Say). Dorsal aspect. Scale bar 1 mm.

**Habitus.** dorsal aspect, as in Fig. 54.

##### Measurements and variation.

Variation of measurements (mm) and ratios for *Semiardistomis propinquus* from Mexico (n=6) are: HL = 0,60–0,62–0,63; PL 1,03–1,14–1,25; PW 1,03–1,23–1,30; EL 3,31–3,47–3,63; EW 1,88–2,01–2,13; SBL 5,04–5,23–5,44; PW/EW 0,61; PW/PL 1,08; PL/EL 0,33; EW/EL 0,58.

Despite the presence of setiferous punctures (other than the usual three in interval 3) and the proportionally larger elytra, this species is markedly similar to *Semiardistomis labialis* and is possibly simply a hirsute form of that species. Similar variation has been documented for other Carabidae, including *Agonum decorum* (Say) (Liebherr 1983).

##### Geographical distribution

(Fig. 59)**.** records indicate that this species is restricted to mountains in southern Mexico.

##### Habitat and activity.

Label data indicate that exemplars of this species have been collected during the night at margins of a small pond.

##### Taxonomic note.

This species probably constitutes a morph of *Semiardistomis labialis* (Chaudoir). If this is shown to be so, the name *Semiardistomis propinquus* will become a junior synonym of the name *Semiardistomis labialis*.

##### Material examined.

In addition to types: MEXICO. Guanajuato (IRSNB, 3) Puebla 20 May 1973 (USNM, 42) Oaxaca Jul. 1964 (USNM, 14) Jalisco S. Guadalajara Jul 1964 (USNM, 4) Michoacan (USNM, 1) Chiapas May 1974 (FSCA, 12) Guerrero Picaya 12 Jun 2004 (CAS, 8) Morelos, Tepoztlan 27 Oct 2004 (CAS, 7).

#### Semiardistomis semipunctatus

(Dejean, 1831)

http://species-id.net/wiki/Semiardistomis_semipunctatus

Fig. 58

Clivina semipunctata Dejean, 1831: 509.
Ardistomis semipunctata (Dejean): Putzeys 1846: 648.
Ardistomus semipunctatus (Dejean): Csiki 1927: 549; Blackwelder 1944: 27.
Ardistomis semipunctatus (Dejean): Lorenz 2005: 146.
Ardistomis (Semiardistomis) semipunctatus (Dejean): Kult 1950: 316.
Semiardistomis semipunctata (Dejean); Erwin 2011: 238.

##### Type material.

Lectotype, here designated, at MHNP, pinned, labeled: green paper handwritten “Buenos Ayres Lacordaire”/ handwritten “ 24”/ “Lectotype Clivina pallipes Dej. Des. S.W. Nichols 1984”/ printed on red paper “Lectotype”.

##### Type locality.

Given by Dejean (1831: 509) as "parties méridionales de Brésil et dans les environs de Buenos Ayres", Brazil and Argentina.

##### Diagnosis.

Body piceous with green bluish reflections. Elytral surface completely smooth, striae continuously impressed in basal 1/8, without or with few punctures, 3 setiferous pores on interval 3. Abdominal sternum VII with 4+4 setiferous punctures.

##### Measurements and variation.

Variation of measurements (mm) and ratios for *Semiardistomis semipunctatus* (n=18) are: HL = 0,46–0,58–0,65; PL 0,93–1,07–1,16; PW 0,95–1,14–1,28; EL 2,25–2,78–3,06; EW 1,38–1,69–1,88; SBL 3,70–4,43–4,86; PW/EW 0,68; PW/PL 1,07; PL/EL 0,39; EW/EL 0,61.

Morphological variation is observed in the elytral punctures. In three different samples from Sta Catarina, Nova Teutonia, Brazil, exemplars with this form are mixed with exemplars of *Semiardistomis deletus* together with intermediate forms.

##### Geographical distribution

(Fig. 58)**.** The known range of this species is confined to a South American area south of the Tropic of Capricorn, extending from southeastern Brazil westward to western Argentina, and south to southern Uruguay.

##### Material examined.

ARGENTINA. Buenos Aires Delta Parana Jan 1943 (IRSNB, 2). BRAZIL. Campos (HECO, 2); Nova Teutonia (IRSNB, 16); Nova Teutona Sta Catarina Nov 1955 (MHNP, 8); Sta Catarina Nov 1970 (USNM, 21). URUGUAY. Montevideo (IRSNB, 4).

#### Semiardistomis viridis

(Say, 1823)

http://species-id.net/wiki/Semiardistomis_viridis

Figs 55 [Fig F18] -57

Clivina viridis Say, 1823: 21; Lindroth and Freitag 1969: 334.
Clivina rostrata Dejean, 1825: 419; Synonymy by LeConte (1857: 80); Lectotype designated by Bousquet (2006: 11).
Ardistomis vicinus Putzeys, 1846: 647; Synonymy by LeConte (1857: 80); Lectotype designated by Bousquet (2006: 11).
Ardistomis rostrata (Dejean): Putzeys 1846: 647.
Ardistomis viridis (Say): LeConte 1857: 80; Putzeys 1866: 214; LeConte 1879: 32; Blatchley 1910: 63; Downie and Arnett 1996: 112.
Ardistomus viridis (Say): Csiki 1927: 549.
Ardistomis (Ardistomiellus) viridis (Say): Kult 1950: 303.
Semiardistomis viridis (Say); Erwin 2011: 238.

##### Type material.

Neotype, designated by Lindroth and Freitag (1969: 334), at Museum of Comparative Zoology, not examined.

##### Type locality.

Philadelphia Neck, Pennsylvania, U.S.A.

##### Diagnosis.

Body piceous with green reflections. Two pairs of supraorbital setae. More than two premedial setiferous punctures on pronotal disc. Elytral surface completely smooth, striae absent, punctures deeply impressed, setiferous. Abdominal sternum VII with 6+6 setiferous punctures. Body length given by Bousquet (2006: 11) as 5.0–6.5 mm.

##### Habitus.

dorsal aspect, as in Fig. 55.

##### Geographical distribution

(Fig. 59)**.** Known from southeastern United States and Bahama Islands in the West Indies (Nichols 1988a; Bousquet 2006).

##### Habitat and activity.

**Figure 56. F18:**
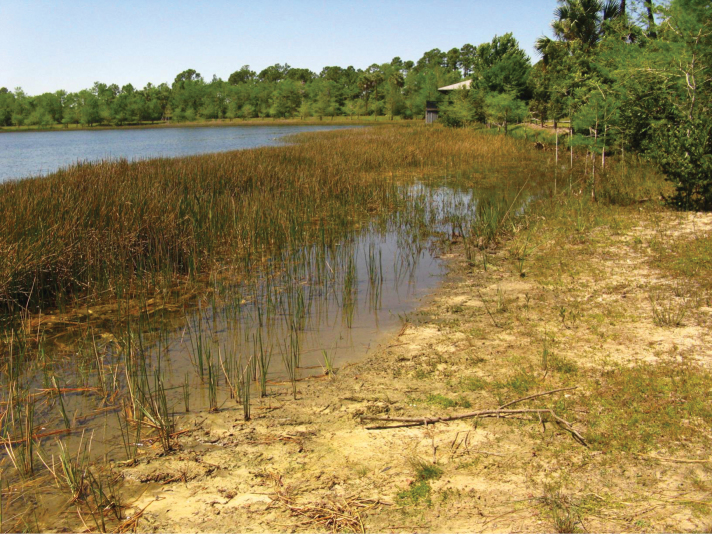
Habitat of *Semiardistomis viridis* (Say) at Grassy Waters Preserve, West Palm Beach, Florida, USA

I collected this species in the “Eagle trail” at the Grassy Waters Preserve in West Palm Beach on April 2, 2011. Adults were active during the day on sandy margins of a fresh water pond (Fig. 56).

##### Material examined.

USA. Marion Co. V 99 Fla (USNM, 2) Jackson Co, Fla XII 97 (FSCA, 4). Sarasota Co, Fla. May 68 (USNM, 2) San Jacinto Co. Tex, Jun 68 (USNM, 4). West Palm Beach, Fla, March 2011(PVCCu, 12) Etats unis (MHNP, 6).

## A zoogeographic scenario

The distribution of the species of the *puncticollis* group is inadequately known because most of the species have been rarely collected (except for *Semiardistomis puncticollis* and *Semiardistomis laevistriatus*). Nonetheless, some patterns can be inferred. The plesiotypic state for some characters seems to be concentrated in the northern portion of South America, with putatively apotypic character states distributed peripherally in a radial fashion. This pattern is similar for the *labialis* species–group. Since both groups show a similar pattern, I postulate a possible center of origin for the genus and then radiation of descendant lineages from, Northern South America.

The northern radiant of the *puncticollis* species-group reached temperate southern North America through what is now Middle America (Central America + Mexico). That lineage became extinct, except for its northern descendant, *Semiardistomis puncticollis*. An eastern lineage colonized the West Indian Lesser Antilles, evidence being the extant *Semiardistomis laevistriatus*, which became a forest-inhabiting brachypterous humicole, confined now to the Island of Guadeloupe (Basse Terre). Incidentally, Guadeloupe houses other clivinines: *Ardistomis atripennis* Putzeys (macropterous) and *Ardistomis guadeloupensis* Kult (brachypterous); and two species of the genus *Oxydrepanus*.

Southward, the distributional record is too incomplete to infer patterns. Notably are localities with many sympatric species, evidencing high power of dispersal in this southern assemblage, being Loreto and Madre de Dios in Peru the localities with highest number of sympatric species.

The *labialis* species–group seems to follow a similar zoogeographic structure. Northward from the putative center of radiation (Fig. 59), *Semiardistomis viridis* is the most derivative form. It is broadly sympatric with *Semiardistomis puncticollis*, and like that species may be the oldest survivor of an interruption of the ranges of the Central American lineages. But here, contrary to *puncticollis* species–group, a second event arose in *Semiardistomis labialis* and related forms in Central America, from which source originated the stock that gave rise to the West Indian Greater Antillean *Semiardistomis cyaneolimbatus*. That stock may have reached the islands by overseas dispersal, or by means of a now foundered land bridge. More recently, the West Indian Bahamas was invaded by *Semiardistomis viridis*, no doubt by overseas dispersal.

Southward (Fig. 58) in South America, , the structurally derivative forms of the *labialis* species– group do not show defined patterns of speciation possibly for several reasons, including the following. First, as far as I can determine, interspecific differentiation is ruled just by some variations in the elytra surface and those variations have shown to be unstable inside localities samples checked. Second, contrary to *puncticollis* species–group, sympatric “species” share every grade of intermediate forms evidencing a continuous flux between populations. Probably gene flow has not been interrupted southward and we just see the result of the polymorphic condition of only one species derivate from a northern ancestor.

In both species–groups some morphological characteristics, like reduction of the prolongation of the median carina of the mentum and hirsutism, have arisen northward from center of radiation following analog ways, suggesting two different paths of evolution to those states under similar evolutionary pressures.

## Checklist of the species names of the genus *Semiardistomis* Kult

**Genus *Semiardistomis* Kult, 1950**

**Species–group *puncticollis***

*Semiardistomis cordicollis* (Putzeys, 1846)

*Semiardistomis darlingtoni* (Kult, 1950)

*Semiardistomis exspectatus* sp. n.

*Semiardistomis glabratus* (Putzeys, 1866)

*Semiardistomis balthasari* (Kult, 1950)

*Semiardistomis jedlickai* (Kult, 1950)

*Semiardistomis laevistriatus* (Fleutiaux & Sallé, 1889)

*Semiardistomis maindroni* (Kult, 1950)

*Semiardistomis major* sp. n.

*Semiardistomis pilosellus* (Kult, 1950)

*Semiardistomis puncticollis* (Dejean, 1831)

*Semiardistomis rugosus* (Putzeys, 1866)

*Semiardistomis subglabra* (van Emden, 1949)

*Semiardistomis vlastae* (Kult, 1950)

**Species–group *labialis***

*Semiardistomis cyaneolimbatus* (Chevrolat, 1863)

*Semiardistomis gundlachi* (Putzeys, 1866)

*Semiardistomis deletus* (Putzeys, 1846)

*Semiardistomis emdeni* (Kult, 1950)

*Semiardistomis flavipes* (Dejean, 1831)

*Semiardistomis aenea* (Putzeys, 1866)

*Semiardistomis brittoni* (Kult, 1950)

*Semiardistomis marani* (Kult, 1950)

*Semiardistomis labialis* (Chaudoir, 1837)

var. *picipes* (Bates, 1881)

var. *nanus* (Bates, 1881)

var. *dilatatus* (Bates, 1881)

*Semiardistomis tuspanensis* (Putzeys, 1846)

*Semiardistomis pallipes* (Dejean, 1831)

var.* caerulea* (Putzeys, 1846)

*Semiardistomis striga* (Putzeys, 1866)

*Semiardistomis propinquus* (Putzeys, 1866)

*Semiardistomis semipunctatus* (Dejean, 1831)

*Semiardistomis viridis* (Say, 1823)

*Semiardistomis vicinus* (Putzeys, 1846)

*Semiardistomis rostrata* (Dejean, 1825)

## Concluding remarks

This revision has added an important part in understanding of the structure of subtribe *Ardistomina*. At this point the road is clear to complete revisions for the rest of Ardistomine genera, which are under construction and will help with new elements to a better comprehension of inter generic relationships. Some questions have to be solved inside the genus originated in part by poor representation in collections of material from many areas mainly from South America. For sure, new samples and DNA studies will contribute to solve unclear specific status. In species–group labialis we have most of problems due to polymorphic condition of most species. In Central America *Semiardistomis propinquus* must be evaluated using consistent genetic data to determinate whether or not it is an isolated species from *Semiardistomis labialis*. In South America we just will be able to corroborate denominations of species *Semiardistomis pallipes*, *Semiardistomis flavipes*, *Semiardistomis deletus* and *Semiardistomis semipunctatus* when gaps in distributional records will filled with new material mainly from Amazonian and Central Brazil and meridional half of Argentina; only them, we will have data enough to picture how different characters with taxonomic value behave in relation with geographic position.

**Figure 57.**
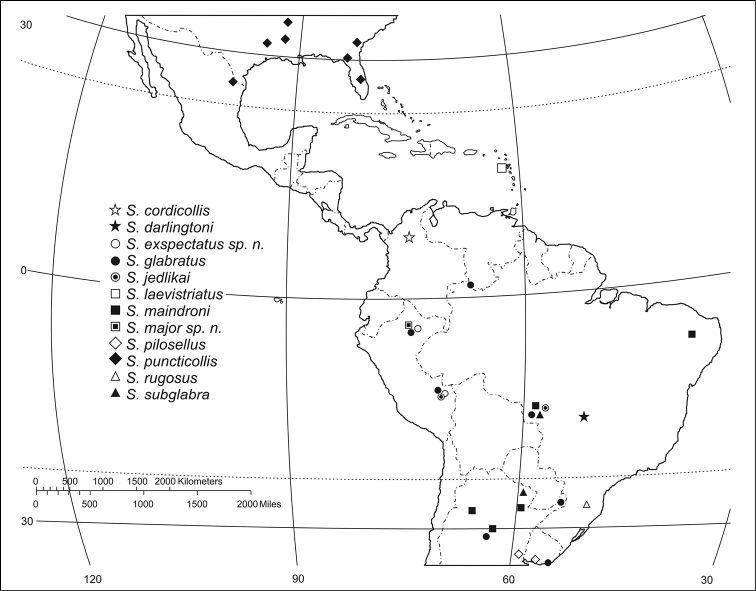
Distribution map of members of the *puncticollis* species–group.

**Figure 58.**
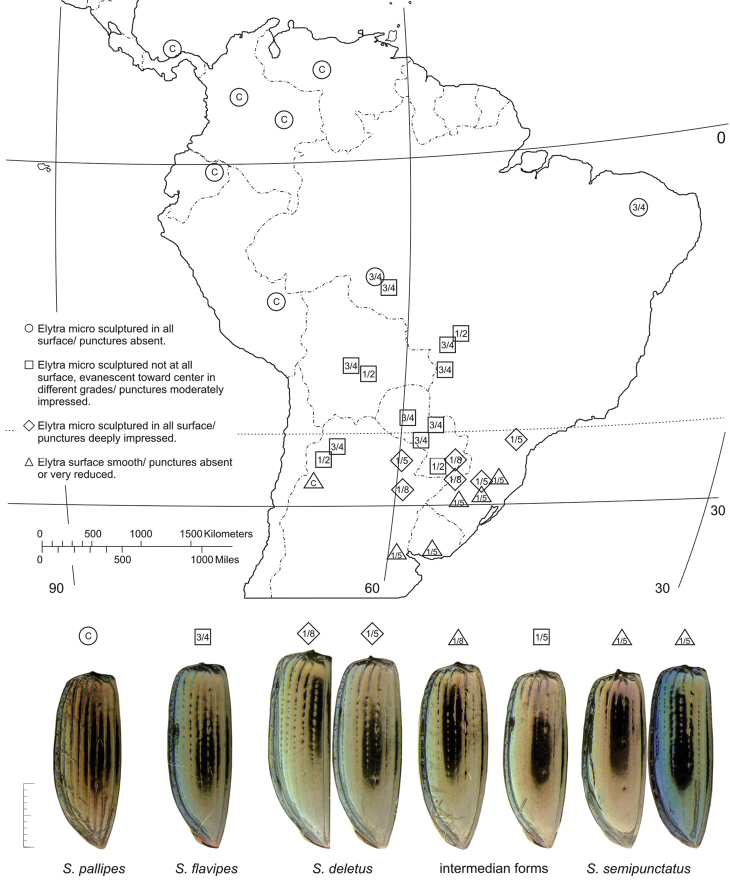
Distribution map, illustrations of exemplar left elytra, and variability of members of the *labialis* species–group in South America. Value inside symbol indicates portion of basal elytra in which striae are impressed, (C, complete impressed). Scale bar 1 mm.

**Figure 59.**
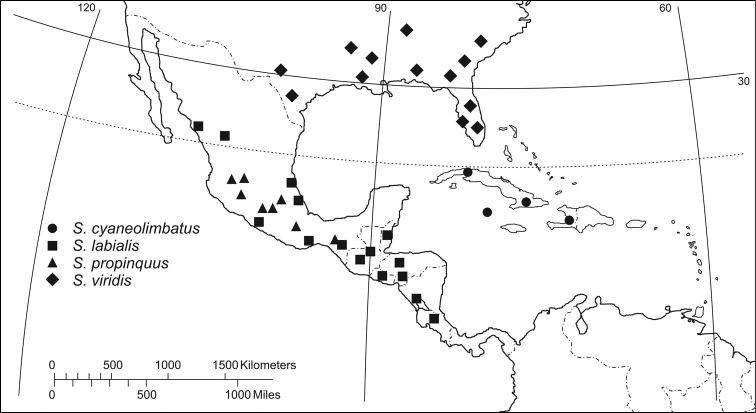
Distribution map of members of the *labialis* species–group in Central America.

## Supplementary Material

XML Treatment for Ardistomina

XML Treatment for Semiardistomis

XML Treatment for Semiardistomis cordicollis

XML Treatment for Semiardistomis darlingtoni

XML Treatment for Semiardistomis exspectatus

XML Treatment for Semiardistomis glabratus

XML Treatment for Semiardistomis jedlickai

XML Treatment for Semiardistomis laevistriatus

XML Treatment for Semiardistomis maindroni

XML Treatment for Semiardistomis major

XML Treatment for Semiardistomis pilosellus

XML Treatment for Semiardistomis puncticollis

XML Treatment for Semiardistomis rugosus

XML Treatment for Semiardistomis subglabra

XML Treatment for Semiardistomis cyaneolimbatus

XML Treatment for Semiardistomis deletus

XML Treatment for Semiardistomis flavipes

XML Treatment for Semiardistomis labialis

XML Treatment for Semiardistomis pallipes

XML Treatment for Semiardistomis propinquus

XML Treatment for Semiardistomis semipunctatus

XML Treatment for Semiardistomis viridis
